# Supplementary material for: ColoPola: A polarimetric imaging dataset for colorectal cancer detection
Source: Gigascience. 2025 Oct 16;14:giaf120. doi: 10.1093/gigascience/giaf120 (PMC12530094; doi:10.1093/gigascience/giaf120)
Supplement: giaf120_GIGA-D-25-00173_Original_Submission [file giaf120_giga-d-25-00173_original_submission.pdf]

GigaScience

ColoPola: A polarimetric imaging dataset for colorectal cancer detection

--Manuscript Draft--

**Manuscript Number:**	GIGA-D-25-00173
**Full Title:**	ColoPola: A polarimetric imaging dataset for colorectal cancer detection
**Article Type:**	Research
**Funding Information:**	Viet Nam National University Ho Chi Minh City (DS2023-28-02) — Assoc. Prof Thi Thu Hien Pham
**Abstract:**	Backgrounds: In recent years, polarimetric imaging has been developed for various biological applications, including tissue morphological characterization and cancer-stage detection. However, to facilitate classification models based on the characteristics of polarization states, it is essential to develop a consistent and standardized dataset of polarimetric images.<br>Findings: This study presents a dataset of colorectal cancer polarimetric images designated as ColoPola, which is intended to facilitate research efforts in the field and is publicly available at https://doi.org/10.5281/zenodo.10068018. The dataset consists of 572 sample slices (288 healthy and 284 malignant). For each slice, 36 polarimetric images corresponding to different polarization states are provided. Thus, ColoPola contains 20,592 polarimetric images, of which 10,368 correspond to healthy samples and 10,224 to malignant samples. To the best of the authors' knowledge, the dataset is the first of its kind for colorectal cancer images. The practical utility of the dataset is evaluated using five models: three models constructed from scratch (CNN, CNN_2, EfficientFormerV2) and two pretrained models (DenseNet and EfficientNetV2). For each model, the input has a size of 224×224×36, corresponding to the width, height, and red channel value of the polarimetric images, respectively.<br>Conclusions: The results show that the CNN, CNN_2, EfficientFormerV2, DenseNet, and EfficientNetV2 models obtain F1 scores of 0.870, 0.862, 0.908, 0.903, and 0.965, respectively, on the testing set. Among the five models, EfficientNetV2 achieves the best performance, with all the performance metrics exceeding 0.95 for both the validation set and the testing set. Overall, the results suggest that ColoPola has significant potential as a polarimetric optical imaging-based diagnostic tool for colorectal cancer in clinical practice.
**Corresponding Author:**	Thi Thu Hien Pham, Ph.D.<br>International University<br>Ho Chi Minh City, Thu Duc VIET NAM
**Corresponding Author Secondary Information:**	
**Corresponding Author's Institution:**	International University
**Corresponding Author's Secondary Institution:**	
**First Author:**	Thi Thu Hien Pham, Ph.D.
**First Author Secondary Information:**	
**Order of Authors:**	Thi Thu Hien Pham, Ph.D.
	Quoc-Hoang-Quyen Vo
	Thao-Vi Nguyen
	The-Hiep Nguyen
	Quoc-Hung Phan
	Thanh-Hai Le
**Order of Authors Secondary Information:**	
**Additional Information:**	

Powered by Editorial Manager® and ProduXion Manager® from Aries Systems Corporation

Question	Response
Are you submitting this manuscript to a special series or article collection?	No
**Experimental design and statistics**<br><br>Full details of the experimental design and statistical methods used should be given in the Methods section, as detailed in our Minimum Standards Reporting Checklist. Information essential to interpreting the data presented should be made available in the figure legends.<br><br>Have you included all the information requested in your manuscript?	Yes
**Resources**<br><br>A description of all resources used, including antibodies, cell lines, animals and software tools, with enough information to allow them to be uniquely identified, should be included in the Methods section. Authors are strongly encouraged to cite Research Resource Identifiers (RRIDs) for antibodies, model organisms and tools, where possible.<br><br>Have you included the information requested as detailed in our Minimum Standards Reporting Checklist?	Yes
**Availability of data and materials**<br><br>All datasets and code on which the conclusions of the paper rely must be either included in your submission or deposited in publicly available repositories (where available and ethically appropriate), referencing such data using a unique identifier in the references and in the "Availability of Data and Materials" section of your manuscript.	Yes

Have you have met the above requirement as detailed in our Minimum Standards Reporting Checklist?	
GigaScience has policies and guidelines in place for the use of generative AI-writing tools such as ChatGPT. If you have used such writing tools to assist with writing the manuscript this must be declared and cited in the text. Authors should not list AI-writing tools and other AI-assisted technologies as an author or co-author and should acknowledge that they are fully responsible for text generated or refined by AI-writing tools.<p><br><br>A summary of use (particularly in the introduction or among methods) needs to be included at the end of the paper, and the outputs should also be included as a supplementary file hosted in GigaDB or other open repositories. Please read our guidelines for more information. <p><br><br>By submitting to GigaScience, you are aware of the journal's AI-writing tools policy, and if you have declared use of such tools below, you have acknowledged this where appropriate in your manuscript and have made a summary of use and outputs available. <p><br><b>AI-assisted writing tools have been used in the preparation of this manuscript?	No

Click here to access/download;Manuscript;Manuscript.docx

ColoPola: A polarimetric imaging dataset for colorectal cancer detection

Thi-Thu-Hien Pham[1,2,*], Quoc-Hoang-Quyen Vo[1,2], Thao-Vi Nguyen[1,2], The-Hiep Nguyen[1,2], Quoc-Hung Phan[3], and Thanh-Hai Le[4,*]

[1] School of Biomedical Engineering, International University, Ho Chi Minh City, Vietnam

[2] Vietnam National University HCMC, Ho Chi Minh City, 700000, Vietnam

[3]Mechanical Engineering Department, National United University, Miaoli 36063, Taiwan

[4]Department of Information Technology Specialization, FPT University, Ho Chi Minh City 700000, Vietnam

*Corresponding author: ptthien@hcmiu.edu.vn, and hailt56@fe.edu.vn

Abstract

Backgrounds: In recent years, polarimetric imaging has been developed for various biological applications, including tissue morphological characterization and cancer-stage detection. However, to facilitate classification models based on the characteristics of polarization states, it is essential to develop a consistent and standardized dataset of polarimetric images.

Findings: This study presents a dataset of colorectal cancer polarimetric images designated as ColoPola, which is intended to facilitate research efforts in the field and is publicly available at https://doi.org/10.5281/zenodo.10068018. The dataset consists of 572 sample slices (288 healthy and 284 malignant). For each slice, 36 polarimetric images corresponding to different polarization states are provided. Thus, ColoPola contains 20,592 polarimetric images, of which 10,368 correspond to healthy samples and 10,224 to malignant samples. To the best of the authors' knowledge, the dataset is the first of its kind for colorectal cancer images. The practical utility of the dataset is evaluated using five models: three models constructed from scratch (CNN, CNN_2, EfficientFormerV2) and two pretrained models (DenseNet and EfficientNetV2). For each model, the input has a size of 224×224×36, corresponding to the width, height, and red channel value of the polarimetric images, respectively.

Conclusions: The results show that the CNN, CNN_2, EfficientFormerV2, DenseNet, and EfficientNetV2 models obtain F1 scores of 0.870, 0.862, 0.908, 0.903, and 0.965, respectively, on the testing set. Among the five models, EfficientNetV2 achieves the best performance, with all the performance metrics exceeding 0.95 for both the validation set and the testing set. Overall, the results suggest that ColoPola has significant potential as a polarimetric optical imaging-based diagnostic tool for colorectal cancer in clinical practice.

Keywords: ColoPola dataset, Colorectal cancer, CNN, DenseNet, EfficientFormerV2, EfficientNetV2, Mueller matrix transformation, Polarimetric imaging.

1. Introduction

Colorectal cancer (CRC) is one of the most common malignancies worldwide and is a leading cause of cancer-related death in both men and women. GLOBOCAN estimated that CRC was the third most common cancer type globally in 2020, accounting for approximately 10% of all cancer cases (including 1.9 million new cancer cases and over 915 thousand deaths) [1]. Hence, there is a requirement for effective diagnostic methods capable of detecting CRC at the earliest stage possible. Colorectal cancer usually originates in the colon or rectum and is classified as colon or rectal cancer accordingly. The majority of CRCs start as a growth on the inner layer of the colon or rectum. These growths, known as polyps, may become cancerous over time (usually 10-15 years); however, not all polyps do [2]. The tumor stage at the time of treatment is the most significant predictor of survival. However, CRC identification in symptomatic individuals is challenging because some of the symptoms of colorectal cancer can be non-specific and may overlap with those of other gastrointestinal conditions.

Colonoscopy and biopsy, together with stool-based diagnostics and visual structural examinations, are considered the "gold standard" for CRC assessment [3]. However, owing to limited endoscopic resources, the high demand for colonoscopies can lead to prolonged waiting periods, potentially delaying the detection of CRC. Thus, computed tomography (CT) colonography is often preferred, especially for seniors with specific symptoms such as stomach pain or weight loss [4, 5]. However, CT colonography is not only

expensive but also raises important concerns regarding radiation exposure, particularly if follow-up testing is required. Many CRC screening methods are available, including stool DNA testing, colonoscopy with biopsy, ultrasound, X-ray, CT, and magnetic resonance imaging (MRI) scanning [6]. Although these methods have reasonable accuracy, they are prone to lost time, false-positive test results, and a high price tag relative to typical patient incomes. Stool DNA testing, in particular, may result in false-positive results, missed polyps and cancers, and the need for three-yearly colonoscopy in the event of abnormalities [7, 8]. Furthermore, while colonoscopy can usually visualize the whole colon and a biopsy can be performed to remove polyps if necessary, it may miss tiny polyps, cause mild bleeding, bowel tears, or infection, and require anesthesia, which may be disruptive to the patient's daily routine. In addition, long-term exposure to X-ray radiation has adverse effects on human health [9, 10]. Therefore, there is an important need for non-invasive in situ techniques capable of detecting CRCs with high accuracy and low cost.

Mueller matrix polarimetry is an effective method for evaluating the microstructure of biological materials and has found widespread use in biomedical sensing. Polarized light has long been used to aid in the imaging of turbid materials. For example, Schmitt et al. [11] investigated the attenuation of linear and circular polarization light as it traveled through a light-scattering medium. Jacques et al. [12] investigated the point-spread function of reflected polarized light in turbid settings and advocated its use in superficial tissue reflectance video imaging. Demos et al. [13] used time-resolved polarized light transport measurements and reflected polarized charge-coupled device (CCD) photography to perform the non-invasive imaging of biological samples. Mourant et al. [14] employed a CCD camera to examine the wavelength dependence of polarized light in normal and malignant cell solutions. The results indicated that the depolarization power of malignant samples was a reliable predictor of the cancer growth stage and histological variety. Thus, several Mueller matrix transformation (MMT) parameters were additionally proposed to provide additional quantitative information on the structural and optical properties of the sample.

As artificial intelligence (AI) technology has advanced in recent years, it has been increasingly applied for the detection and diagnosis of many cancers, including CRC [15−21]. Kim et al. [15] developed a Hessian matrix-based computer-aided detection (CAD) algorithm to localize potential cancerous polyps in CT colonography images. Chen et al. [16] used a deep neural network (DNN) to analyze narrow-band images of diminutive colorectal polyps in a dataset of 1476 images of neoplastic polyps and 681 images of hyperplastic polyps. The network achieved a classification accuracy of 90.1% and a sensitivity of 96.3%. Thakur et al. [17] conducted a systematic review of the use of machine learning models in the analysis of CRC pathology images. The results showed that deep learning models such as DCAN, CNN, U-Net, and FCN achieved a good segmentation performance when applied to gland segmentation in the Warwick-Qu and CRAG datasets for both benign and malignant tissue samples. Iizuka et al. [18] evaluated the performance of several deep-learning models when applied to tumor classification, microenvironment analysis, and prognosis prediction tasks for CRC images. It was shown that convolutional neural networks (CNNs) and recurrent neural networks (RNNs) trained on biopsy histopathology whole-slide images of the stomach and colon achieved area under the receiver operating characteristic curves (AUCs) of up to 0.96 for colonic adenocarcinomas. Xu et al. [19] compiled a dataset of 85 normal colorectal tissue slides and 222 colorectal cancer tissue slides from Hematoxylin and Eosin (H&E)-stained tissue sections. An InceptionV3 model was trained using a transfer learning technique and was used to segment the tumor regions in the images. The proposed model achieved an average accuracy of 0.936 and a Dice score of 0.885 for cancerous slices. Yu et al. [20] proposed a semi-supervised learning (SSL) algorithm based on a mean teacher architecture and evaluated the model on 13,111 histological CRC images acquired from 8803 subjects. The results showed that the proposed method achieved an AUC performance similar to that of a supervised learning (SL) method while requiring significantly less labeled data. Tharwat et al. [21] reviewed the effectiveness of various machine learning (ML) and deep learning (DL) techniques in performing the early-stage detection of CRC. The strengths and limitations of the different methods were identified, and opportunities for future research on the automatic diagnosis of colon cancer were proposed.

Overall, the above studies [15-21] confirm the feasibility of combining polarimetry and artificial intelligence frameworks to perform automatic diagnosis and classification of CRCs. However, to the best of the authors' knowledge, polarized images of CRC tissues have not yet been published in the image processing community. To address this gap, this study introduces a dataset of CRC polarimetric images, designated as ColoPola, which comprises optical images of normal and colorectal cancer tissue samples acquired using a Mueller matrix polarimetry technique. The ColoPola dataset not only fills a critical gap in available biomedical imaging data but also sets a new standard for the early detection of CRC, offering a potential reduction in the reliance on invasive procedures and improving the prognosis for patients through earlier intervention. The practical utility of the dataset is assessed using five ML models (CNN, CNN_2, EfficientFormerV2, DenseNet, and EfficientNetV2). A novel data input is generated in which the red channels of the 36 polarimetric color images are concatenated for each sample. The experimental results confirm that ColoPola has considerable promise as a non-invasive, optical imaging-based diagnostic tool for colorectal cancer in clinical settings.

2. Sample preparation

Five hundred and seventy-two slices of healthy and colorectal cancer tissue were acquired from the pathology departments of Binh Duong Provincial General Hospital in Binh Duong Province and the 115 People's Hospital in Ho Chi Minh City, both located in Vietnam. The slices were provided with patient consent, and all the treatments and experiments were performed following relevant guidelines and regulations. All personal information about these samples were concealed and the sample was then classified by histopathologists. The formol-immersed tissues were kept at room temperature and analyzed within 72 h of receipt. The tissue samples were sectioned with a microtome at a thickness of 5 μm and placed on 5-mm-thick quartz slides for subsequent analysis. As illustrated in Fig. 1(A), each tissue sample was sliced along the xOz, xOy, and yOz planes to ensure that all the structures within the sample were clearly visible during the polarization imaging process. Figures 1(B) and (C) show a typical stained slide

used for histopathological analysis through a microscope and an unstained slide used for measurement by the polarized light system.

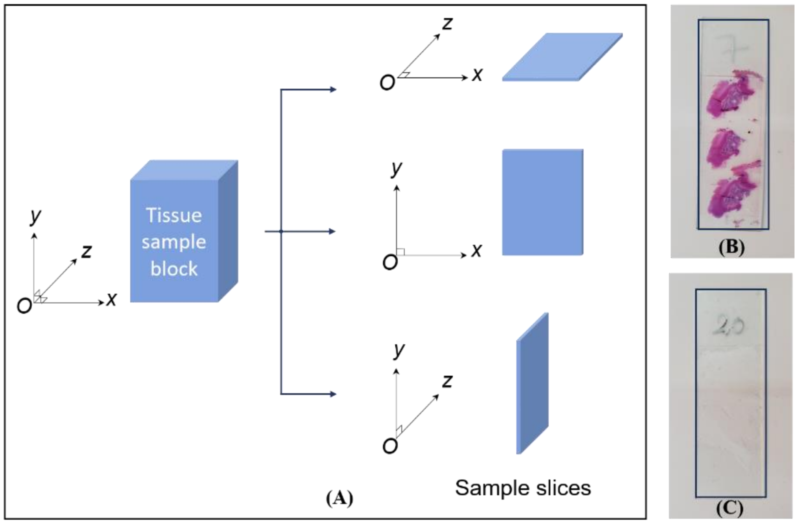

Fig. 1. Sample slices (A) different slice planes from tissue sample block; (B) slice stained with H&E for histological examination; and (C) unstained slice for Mueller matrix imaging measurement.

Fig. 2 shows the sample preparation procedure used in this study. The slices cut in the three directions were mounted on quartz slides for observation and measurement purposes. The stained samples were observed using a microscope to perform conventional histopathological analyses. The unstained samples were placed in a self-built transmission Mueller matrix polarimetry system, where 36 images were obtained by a CCD camera for each sample under different polarization conditions. For both types of samples (stained and unstained), the measurement process was performed at least three times for each slice to ensure the reliability of the observation/measurement results.

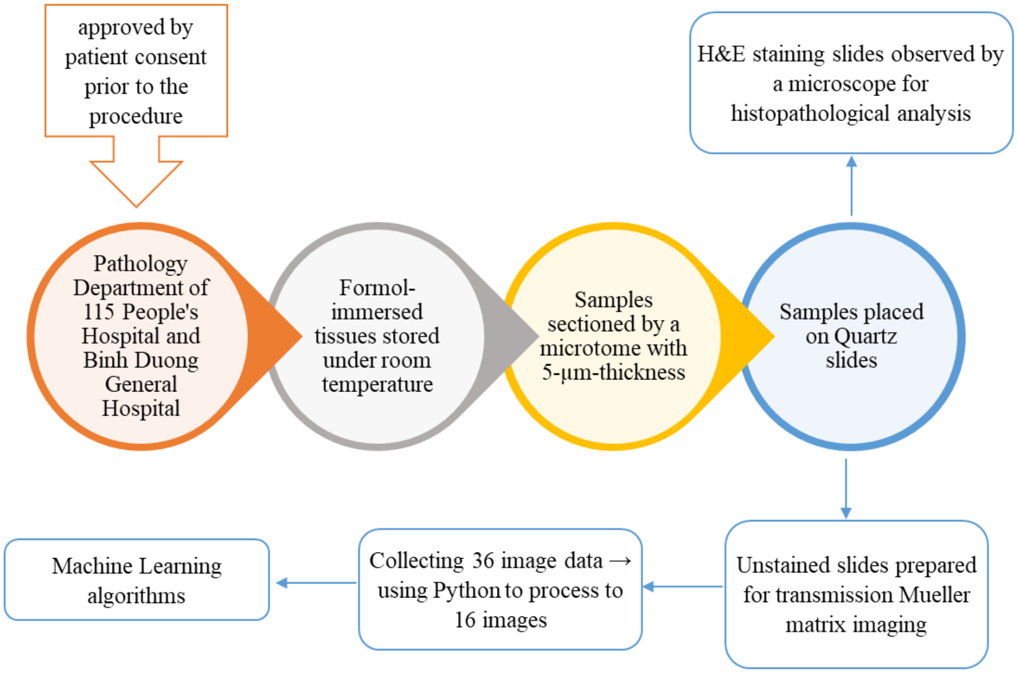

Fig. 2. Sample preparation and basic experimental procedure.

2.1. Histopathological analysis

To serve as a benchmark for the classification results obtained from the Mueller matrix imaging system and deep learning models, the stained H&E samples were carefully annotated by an experienced histopathologist. Fig. 3 presents two images showing the typical histopathological features of rectal cancer [22]. Both images show the disappearance of the normal glandular architecture typical of rectal cancer tissue, together with significant nuclear atypia with protruding nucleoli, and a high nucleus-to-cytoplasm ratio. Both tissues also show high-grade mucinous adenocarcinomas and malignant epithelial cells in clumps and layers in pools of extracellular mucin.

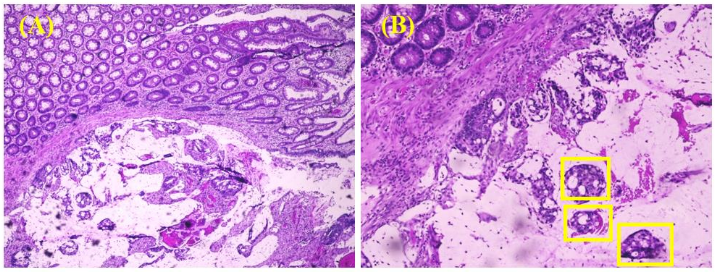

Fig. 3. H&E-stained rectal cancer tissues: (A) Magnification 40X; (B) Magnification 100X.

3. Construction of ColoPola dataset

3.1 Mueller matrix transformation

The Mueller matrix *M* used to define the polarization characteristics of biomedical samples has the form of a 4×4 matrix, in which the elements are obtained using different combinations of polarized light produced by the generator and analyzer modules in the polarimetry system. The matrix has the form [23]

$$M = \begin{bmatrix} m_{11} & m_{12} & m_{13} & m_{14} \\ m_{21} & m_{22} & m_{23} & m_{24} \\ m_{31} & m_{32} & m_{33} & m_{34} \\ m_{41} & m_{42} & m_{43} & m_{44} \end{bmatrix}$$
$$= \begin{bmatrix} HH+HV+VH+VV & HH+HV-VH-VV & PH+PV-MH-MV & RH+RV-LH-LV \\ HH-HV+VH-VV & HH-HV-VH+VV & PH-PV-MH+MV & RH-RV-LH+LV \\ HP-HM+VP-VM & HP-HM-VP+VM & PP-PM-MH+MM & RP-RM-LP+LM \\ HR-HL+VR-VL & HR-HL-VR+VL & PR-PL-MR+ML & RR-RL-LR+LL \end{bmatrix} \quad (1)$$

where six different polarization states of the incident light are generated for measurement purposes: horizontal linear (H), vertical linear (V), 45-degree linear (P), 135-degree linear (M), right circular (R), and left circular (L). From these sox polarization states, a total of 36 images are obtained by the CCD placed after the analyzer module sample, namely *HH*, *HV*, *HP*, *HM*, *HR*, *HL*, *VH*, *VV*, *VP*, *VM*, *VR*, *VL*, *PH*, *PV*, *PP*, *PM*, *MH*, *MV*, *MP*, *MR*, *ML*, *MM*, *RH*, *RV*, *RP*, *RM*, *RR*, *RL*, *LH*, *LV*, *LP*, *LM*, *LR*, and *LL*. Note that the first letter of each notation describes the polarization state generated by the polarization state generator (PSG) in the polarimetry system, while the second letter describes the polarization state generated by the polarization state analyzer (PSA). Having obtained the 36 images, the elements of *M* are constructed by superimposing the images as required. For example, element m_{11} is obtained by superimposing (*HH*), (*VV*), (*HV*), and (*VH*) as $m_{11}=HH+HV+VH+VV$.

The microstructural properties of the tissue samples can be determined using the following Mueller matrix transformation (MMT) parameters [24–29]:

Anisotropy (A)

$$A = \frac{2\left(m_{22}+m_{33}\right)\sqrt{\left(m_{22}-m_{33}\right)^2+\left(m_{22}+m_{33}\right)^2}}{\left(m_{22}+m_{33}\right)^2+\left(m_{22}-m_{33}\right)^2+\left(m_{23}+m_{32}\right)^2}, \in \left[0,1\right] \quad (2)$$

Depolarization power factor (b)

$$b = \frac{m_{22}+m_{33}}{2} \quad (3)$$

Magnitude of anisotropy attribute

$$t = \frac{\sqrt{\left(m_{22}-m_{33}\right)^2+\left(m_{23}+m_{32}\right)^2}}{2} \quad (4)$$

Degree of anisotropy or isotropy

$$G = \sqrt{1-\frac{2(\mathrm{m}_{22}\, m_{33}-\mathrm{m}_{22}\, m_{33})^2}{2(\mathrm{m}^2{}_{23}+m^2{}_{22}+\mathrm{m}^2{}_{33}+m^2{}_{32})^2}} \quad (5)$$

Depolarization power

$$\Delta = 1-\frac{\left|m_{22}\right|+\left|m_{33}\right|+\left|m_{44}\right|}{3}, 0 \le \Delta \le 1 \quad (6)$$

3.2 Image acquisition system

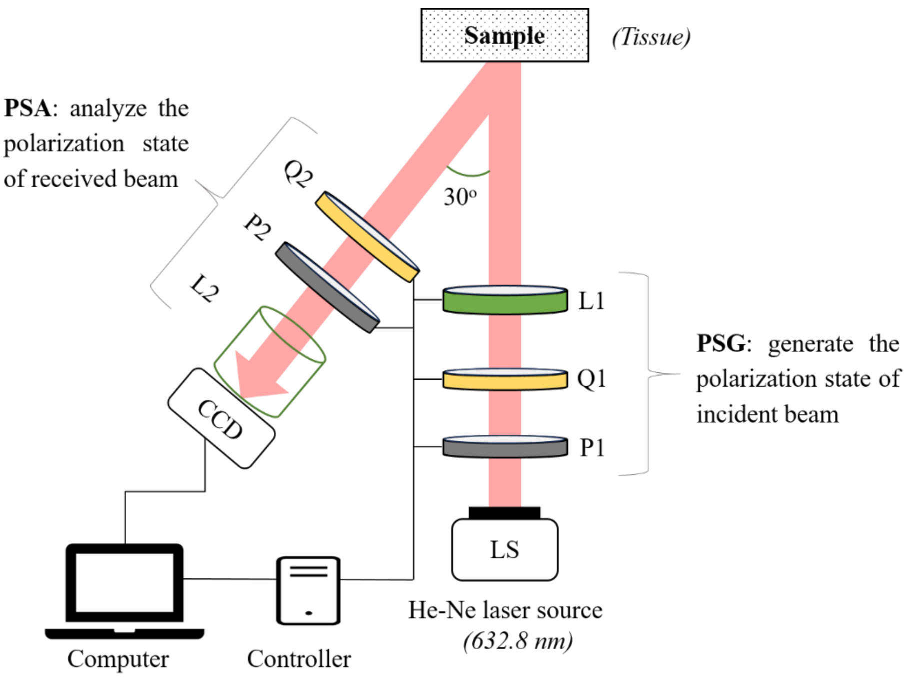

Fig. 4. Schematic of experimental setup.

Figure 4 illustrates the experimental polarimetry system used to obtain the MMT parameters of the healthy and cancerous tissue samples. As shown, the polarization system comprised a PSG block for generating polarized images and a PSA block for analyzing these images. In particular, the PSG block was used to produce incident lights with particular states of polarization, while the PSA block was used to adjust the polarization state of the beam scattered by the sample. The PSG block consisted of a frequency-stable He-Ne laser (HNLS008R, Thorlabs Co.) with a central wavelength of 633 nm, a linear polarizer P1 (GTH5M, Thorlabs Co.) to generate four linear polarization states (i.e., 0° (denoted as *H*), 45° (denoted as *P*), 90° (denoted as *V*), and 135° (denoted as *M*)), a quarter-wave plate Q1 (QWP0-63304-4-R10, CVI Co.) to produce left-handed circular polarization light (denoted as *L*) and right-handed circular polarization light (denoted as *R*), a convex lens L1 (LSSB04-A, Thorlabs, Inc.) and a concave lens L2 (LSSB04-A, Thorlabs, Inc.). The PSA block consisted of a linear polarizer P2 (GTH5M, Thorlabs Co.), a quarter-wave plate Q2 (QWP0-63304-4-R10, CVI Co.), and a CCD camera (CCD, DCU224C, Thorlabs, Inc.) fitted with a zoom lens and connected to a computer. Elements P2 and Q2 in the analyzer performed the same functions as P1

and Q1 in the generator. The polarizers P1 and P2 and quarter-wave plates Q1 and Q2 were mounted on rotation motorized stages (SGSP-60YAW-0B, Sigma Koki Co.) to generate the 36 polarization states required to construct the Mueller matrix for each sample. In the experiments, the linear polarization states of the PSG block were produced by rotating the polarizer (P1), and the circular polarization states were generated by rotating Q1 to the right- and left-hand circular polarization states, respectively. The same procedure was adopted to generate the required polarization states for the PSA block.

Figure 5 illustrates the overall framework of the data collection and analysis tasks performed in this study. As described in Section 2, some of the sliced samples were stained with H&E and observed under a microscope for reference purposes. Meanwhile, the unstained samples were measured using the experimental polarimetry system. The 36 polarization state images captured by the measurement system for each sample were used to construct a colorectal cancer polarimetric (ColoPola) dataset. The polarization images in the ColoPola dataset were used in two ways: (1) to construct Mueller matrix images of the cancerous and healthy tissue samples and analyze the properties (average intensity, polarization parameters, and frequency distribution histograms (FDHs)) of the 16 elements in each sample class; and (2) to serve as the inputs for AI models designed to classify the samples as either healthy or cancerous colorectal tissue.

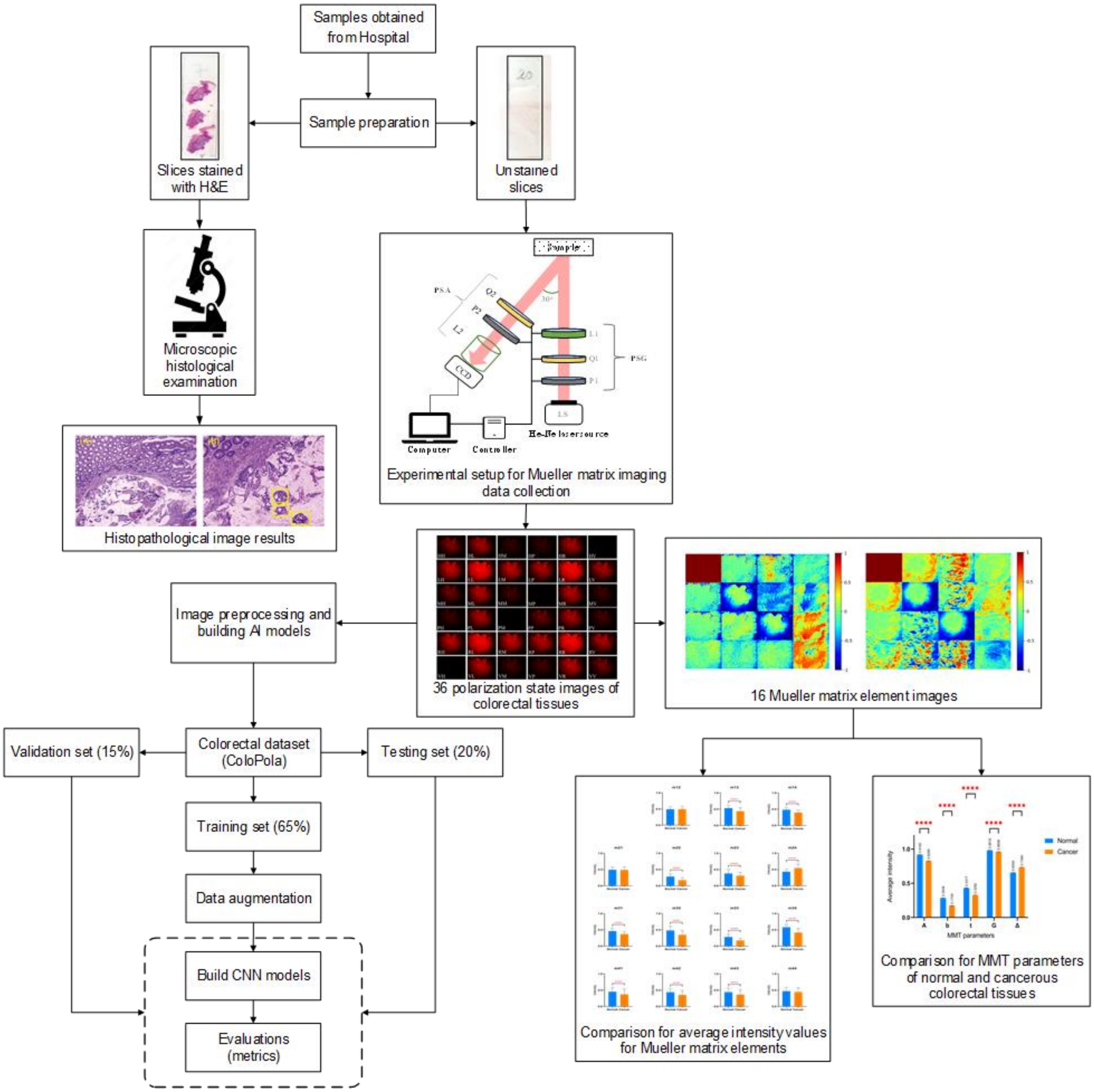

Fig. 5 Research framework.

3.3 ColoPola dataset

Figures 6(A) and 6(B) show the 36 images of a typical healthy tissue sample and a typical malignant CRC sample, respectively, where these images were acquired using six input polarization states and six output polarization states, as described in Section 3.1. The ColoPola dataset consists of 572 tissue slices,

of which 284 are cancer samples and 288 are normal (healthy) samples. For each slice, 36 polarimetric images are prepared. Thus, the dataset contains 20,592 images (10,224 malignant and 10,368 malignant). Each image has a size of 1280×1024 pixels and is stored in the TIFF file format. The dataset is available for download in two RAR files, one containing polarimetric images of normal samples (11.3 GB) and one containing polarimetric images of colorectal cancer samples (14.2 GB). Moreover, a README file (README.md) provides additional information about sample name (name id), alongside two text files (train.txt and test.txt) contain the list of samples in training set (457 samples) and testing set (115 samples) [30].

For the AI classification task, the ColoPola dataset was divided into three sets for training, validation, and testing purposes, respectively, in a ratio of approximately 65:15:20. The size and composition of each set are listed in Table 1. The training set contained 365 slices (184 normal slices and 181 cancer slices). The validation set contained 92 slices (46 normal and 46 cancerous) and the testing set contained 115 slices (58 normal and 57 cancerous). It should be noted that the samples in the ColoPola dataset were uniformly distributed between the normal and cancer classes (288:284 samples) and were randomly assigned to the training, validation, and testing sets.

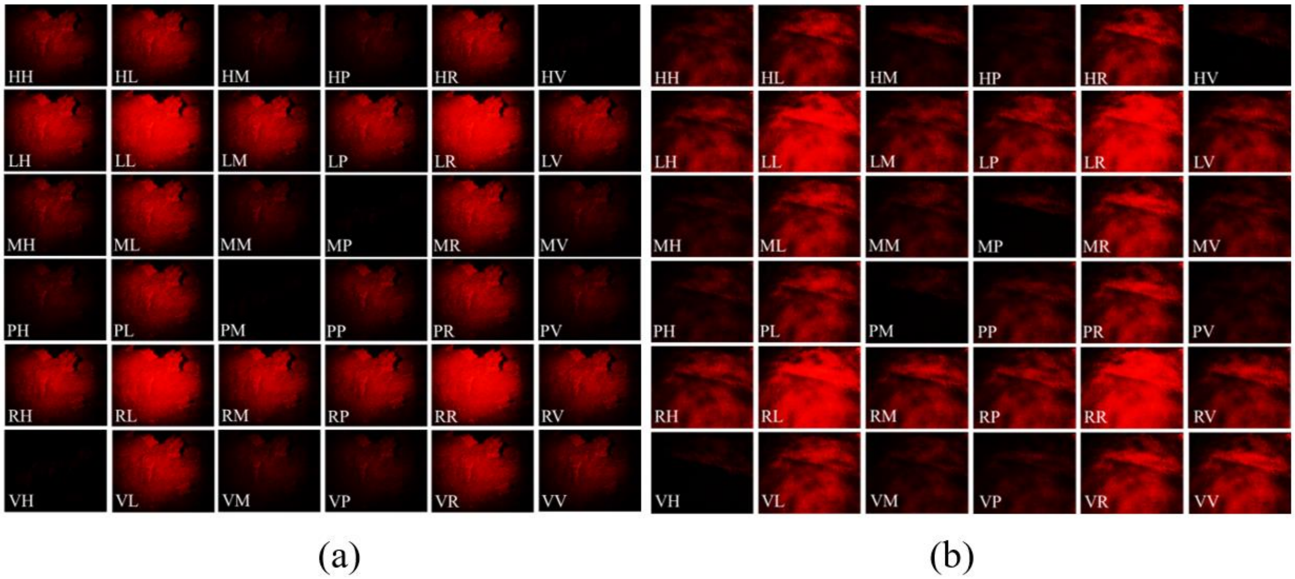

Fig. 6. 36 images of (a) normal and (b) malignant colorectal samples

Table 1 Size and composition of main ColoPola dataset and training, validation, and testing sets.

Type of sample	Specimen (Sample slices)	Image	Training	Validation	Testing
Normal	288	10,368	184	46	58
Cancer	284	10,224	181	46	57
Total	***572***	***20,592***	***365***	***92***	***115***

4. Data Processing and Deep Learning Models

4.1 Data processing

The images captured by the CCD camera had a size of 1280×1024 pixels (Fig. 7). To reduce the computational cost while preserving sufficient polarimetric information for classification purposes, the images were cropped using a kernel of size 900×900 pixels, located at the center of the original image [31]. The images were then saved in the ColoPola dataset in a PNG format.

As described above, the ColoPola dataset contained 36 polarimetric images for each cancerous and healthy sample. Each image consisted of three basic channels: red, green, and blue. Fig. 8 shows the distribution of the intensity values of the 36 color images associated with a typical healthy sample. The intensity values of the green channel are all almost equal to zero, and most of the pixels in the blue channel have intensity values in the interval [0, 4]. In contrast, the intensity values of the red channel vary over the full interval of [0, 255] in most of the 36 images. Fig. 9 shows the frequency distributions of the three color channels for a typical malignant sample. As shown, the color channel information is available for first column (*HH*, *LH*, *MH*, *PH*, *RH*, and *VH*) and fourth column (*HP*, *LP*, *MP*, *PP*, *RP*, and *VP*) of the images. However, for the rest images, no color channel information is available. These images yield no useful information for model training and may introduce errors. Thus, to ensure a consistent learning performance across the two classes (healthy and malignant), the red channel was chosen as the primary input data for each image in the dataset. Accordingly, the size of the input data was set as 900×900×36, corresponding to the width, height, and red channel value of 36 polarimetric images, respectively. Note, however, that in

accordance with the normal input size for most common DL models, the input images were rescaled to a size of 224×224×36 before processing.

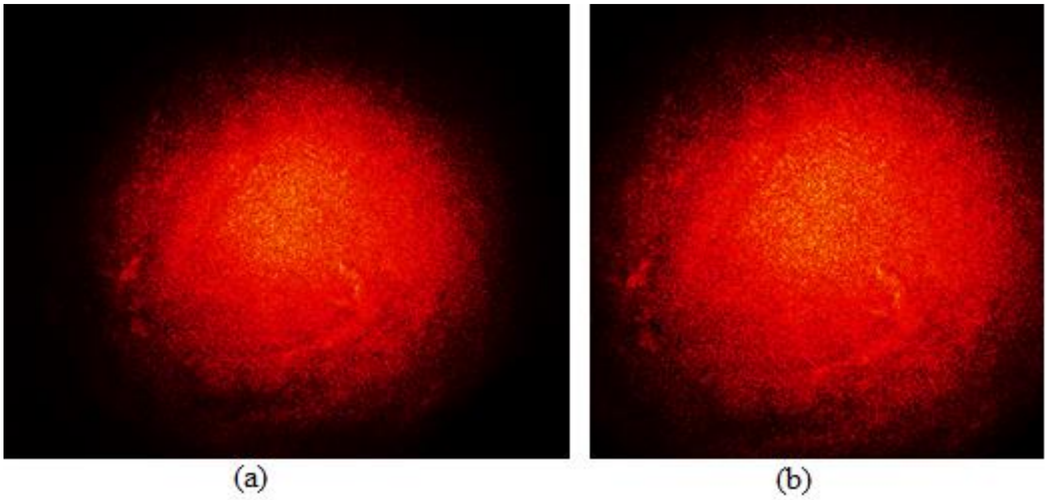

Fig. 7. Input images (a) before and (b) after cropping.

10

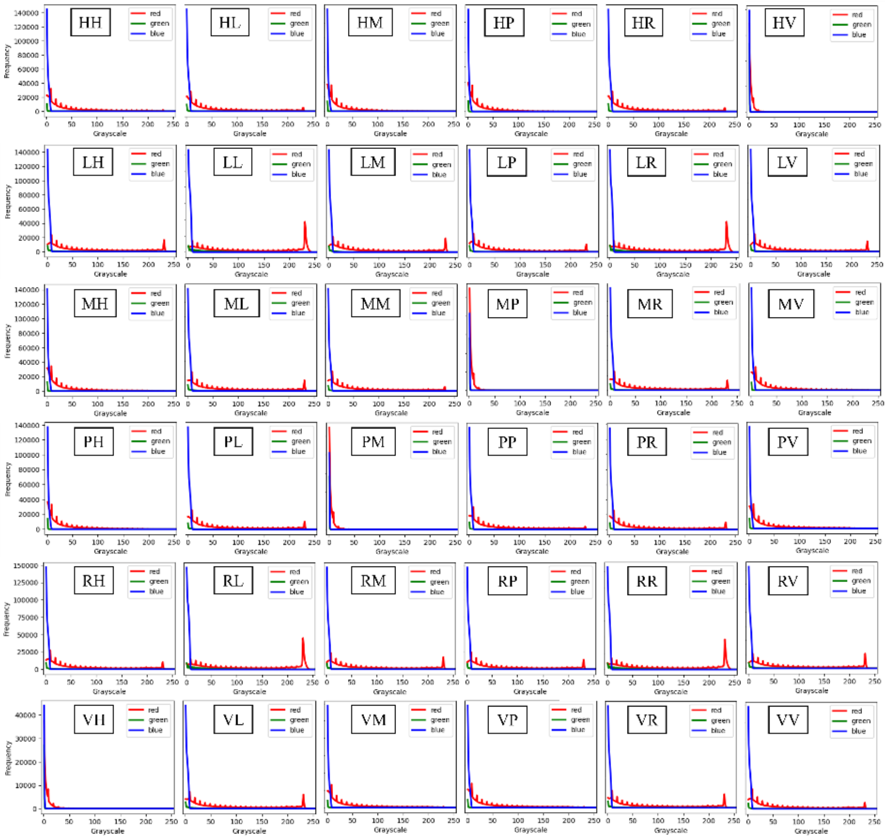

Fig. 8. Histograms of RGB intensity values for normal tissue samples.

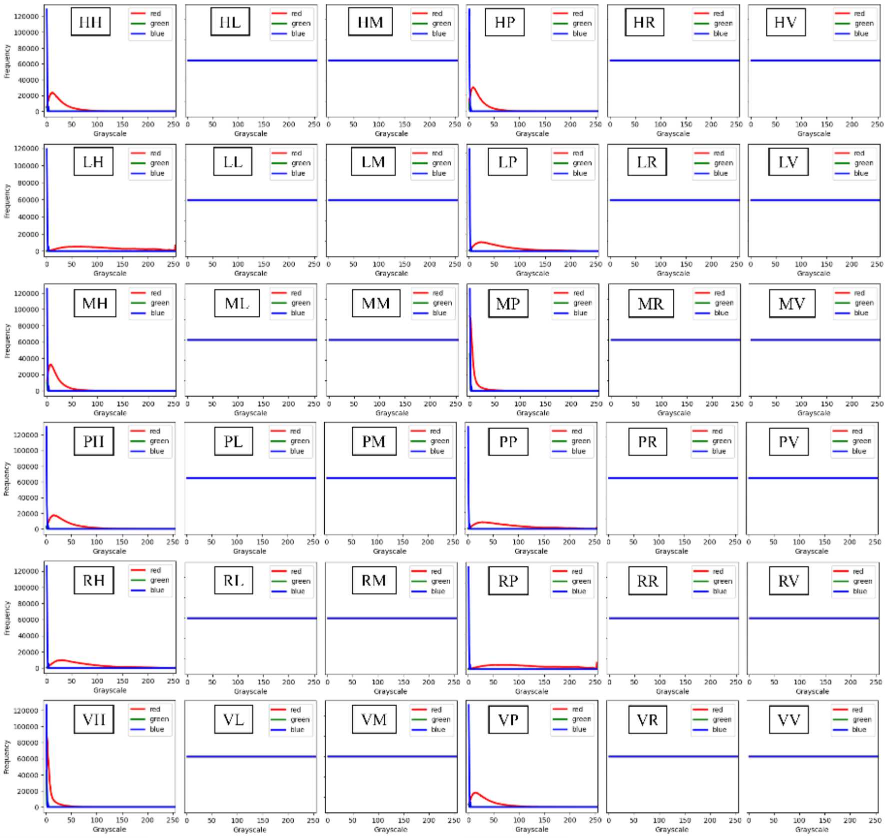

Fig. 9. Histograms of RGB intensity values for malignant tissue samples.

4.2 Deep learning models

The normal and cancerous tissue samples were classified using five deep learning models, including three models built from scratch (i.e., CNN, CNN_2, EfficientFormerV2) and two pretrained models (i.e., DenseNet-121 and EfficientNetV2-M). Fig. 10 shows the architectures of the CNN and CNN_2 models. Both models utilize convolutional blocks consisting of a convolutional layer (Conv) with a kernel size of 3, Batch Normalization (BN) [32], Rectified Linear Unit (ReLU), and Dropout [33] with a probability of 0.2 (after numerous trial-and-error steps). The CNN model has an architecture similar to that of VGG [34], but is smaller. Furthermore, it uses only one convolutional layer before applying an activation function (AvgPool), and then two fully connected (FC) layers to reduce the number of features from 512 to 256. In the CNN_2 model, a convolutional block with a stride of two is used instead of the max pooling layer (MaxPool) to reduce the data dimensions [35]. In addition, a convolutional block with a kernel size of 1×1 is used to replace the FC layers in the CNN model for the same purpose. Meanwhile, the

EfficientFormerV2-S0 model [36] was chosen and trained from scratch, similar to the CNN, CNN_2 models. The EfficientFormerV2 architecture was introduced as a vision transformer and maintain the small size with low latency and high parameter efficiency. The EfficientFormerV2 network was applied various advanced techniques (i.e., token mixer, improved multi-head self-attention, stride attention, and attention on downsampling) for improvements and then utilized a fine-grained joint search method to find the optimal model size and speed. This network outperformed the previous EfficientFormer [37] with similar latency and parameters on several experiments.

Two pretrained DL models, DenseNet-121 [38] and EfficientNetV2-M [39], were selected and fine-tuned for the ColoPola dataset. The DenseNet architecture uses the concept of residual connections, in which all the previous features are concatenated iteratively. By fine-tuning the model in each layer based on all the preceding feature maps, DenseNet can learn the parameters more efficiently. Meanwhile, EfficientNetV2 is optimized to increase the training speed and parameter efficiency. Similar to EfficientNet [40], the EfficientNetV2 model includes several new convolutional blocks (such as Fused-MBConv) that replace the depth-wise 3×3 Conv and expansion 1×1 Conv in EfficientNet with a normal 3×3 Conv after using the neural architecture search. Moreover, the progressive learning with adaptive regularization is applied to gradually increase image size and the regularizations at a specific stage. By doing so, EfficientNetV2 achieves both a faster speed and a smaller size than EfficientNet.

The inputs of the DenseNet-121 and EfficientNetV2-M models both have three channels (i.e., the red, green, and blue color values of the images). However, the inputs of the present study have 36 channels (i.e., 36 polarimetric images of each sample). Therefore, the number of input channels of the first convolutional layer in both models was increased from 3 to 36, where the weights of the first three channels were unchanged while those of the remaining thirty-three channels were initialized using the He technique [41].

Table 2 shows the hyperparameters used to train the five models. As shown, most of the hyperparameters were the same for all five models. However, different initial learning rates were applied to the models built from scratch (CNN, CNN_2 and EfficientFormerV2-S0) and the pretrained models

(DenseNet-121 and EfficientNetV2-M). In particular, CNN and CNN_2 were trained with a higher learning rate to accelerate the model update in the first few epochs. For all five models, learning rate scheduling (ReduceLROnPlateau scheduler) was applied when the metrics ceased to improve in successive iterations during the latter stages of the training process. Moreover, the early stopping technique [42] was utilized to mitigate overfitting by monitoring the validation loss. All of the models were implemented on a desktop computer using the PyTorch library with an Intel i7-12700 CPU, 32 GB RAM, and a GeForce RTX 4070 GPU.

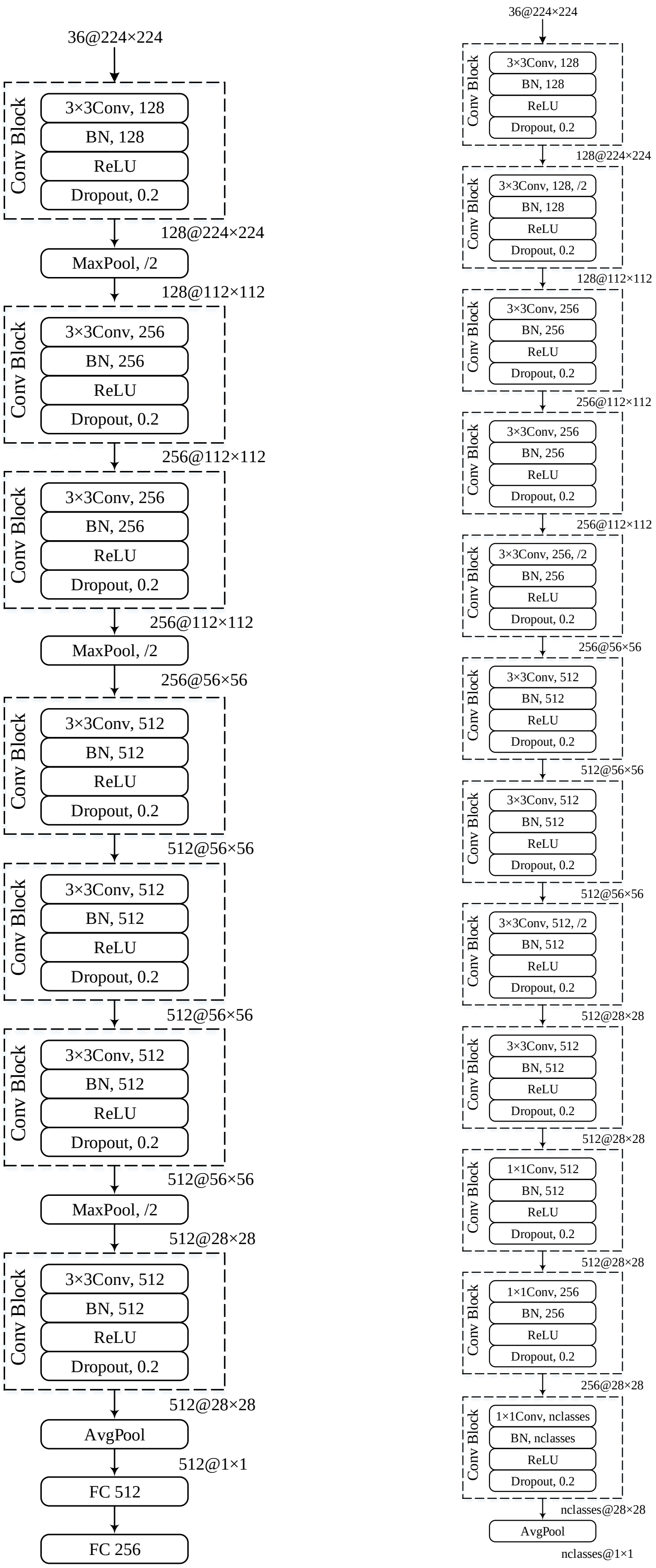

Fig. 10. CNN and CNN_2 architectures.

Table 2. Training hyperparameters

**Parameter**	**Models from scratch**	**Pretrained models**
Optimizer	AdamW [43]	
Batch size	16	
Epoch	200	
Initial learning rate	0.01	0.001
Weight decay	0.001	
Loss	Binary cross entropy loss	

For each model, 572 samples were input into the learning algorithm, where 457 samples were used for training and validation purposes (i.e., 80% of the dataset) and 115 samples were retained for testing (i.e., 20% of the dataset). To increase the amount of training data, an augmentation technique (e.g., random rotation, CLAHE, blur, ...) was applied before the training process (see Fig. 5) [44].

4.3 Performance metrics

The performance of the five classifiers in the training, validation, and testing stages was evaluated using four metrics, namely the accuracy, precision, recall, and F1 score, defined respectively as

$$\text{Accuracy} = \frac{TN + TP}{TN + FP + TP + FN} \tag{7}$$

$$\text{Precision} = \frac{TP}{TP + FP} \tag{8}$$

$$\text{Recall} = \frac{TP}{TP + FN} \tag{9}$$

$$\text{F1score} = 2 \times \frac{\text{Precision} \times \text{Recall}}{\text{Precision+Recall}} \tag{10}$$

where TP, TN, FP, and FN denote true positive, true negative, false positive, and false negative, respectively. The accuracy metric is simply the ratio of the correctly predicted observations to the total number of observations and is thus the most intuitive performance measure. The precision metric evaluates the proportion of positive class predictions that truly belong to the positive class, while the recall metric evaluates the number of positive class predictions as a proportion of the total number of positive examples in the dataset. Finally, the F1 score provides a weighted average of the precision and recall metrics in a single measure.

5. Results and Discussion

5.1. Construction of Mueller matrix images using ColoPola dataset

5.1.1 Mueller matrix images and intensity values

To demonstrate the utility of the ColoPola dataset, this section describes two Mueller matrix images (one for a healthy tissue sample and one for a malignant sample) constructed using the polarization images in the dataset and Eq. (1). Details on the way to convert from 36 polarization state images (Fig. 6) to 16 elements of the Mueller matrix images are publicly available at https://doi.org/10.24433/CO.7469965.v1 (see Ref. [45]). A preliminary investigation revealed that the standard deviations of the element intensities in each sample class (healthy and malignant) were statistically insignificant. Thus, it was inferred that any sample could be used to represent the entire class. For both matrixes in Fig 11, the matrix elements are normalized by m11. Furthermore, the intensity of the Mueller matrix elements has a value in the range of [-1, 1], corresponding to a color change from blue to red. It is seen that the matrixes corresponding to the healthy and malignant samples are qualitatively different. For example, the matrix of the normal colorectal tissue sample is predominantly green, corresponding to a neutral intensity, and the color boundaries between adjacent images are relatively indistinct. By contrast, for the cancerous sample, most of the images are readily distinguishable from their neighbors, and the matrix contains a greater distribution of red and blue pixels, indicating the presence of regions of extreme intensity variation. Overall, the results confirm

the feasibility of using the polarization images in the ColoPola dataset to qualitatively distinguish between healthy and cancerous colorectal tissue samples.

Table 3 lists the intensity values of the Mueller matrix elements for the healthy and malignant colorectal tissues. Both classes show diagonal symmetry, in which elements m_{22} and m_{33} have similar values of 0.2845 and 0.2818, respectively, for the healthy tissue and 0.1732 and 0.1743, respectively, for the malignant tissue. In general, the intensity values of the elements in the cancerous sample are much lower than those in the cancerous sample and show a greater variation across the matrix elements. Thus, it is inferred that the cancerous sample is anisotropic, implying that it has a more complex microstructure.

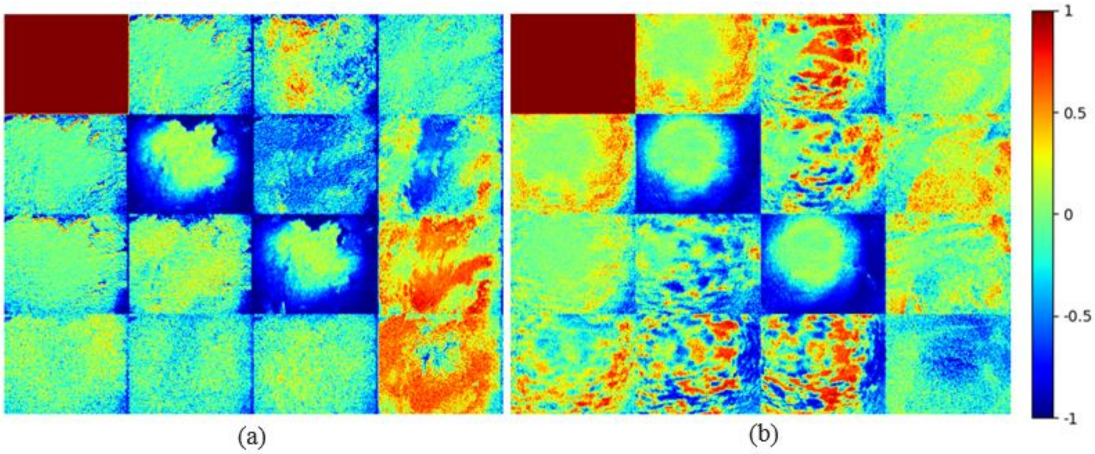

Fig. 11. Mueller matrix images of (a) normal colorectal tissue, (b) cancerous colorectal tissue

Table 3. Average intensity values for each Mueller matrix element in normal and cancerous colorectal tissues.

	m_{11}	m_{12}	m_{13}	m_{14}
**Normal**	1 (normalization)	0.4911 ± 0.0258	0.5327 ± 0.0043	0.4831 ± 0.0055
**Cancer**	1 (normalization)	0.4928 ± 0.0350	0.4361 ± 0.0166	0.3971 ± 0.0375
	m_{21}	m_{22}	m_{23}	m_{24}
**Normal**	0.4934 ± 0.0027	0.2845 ± 0.0087	0.3809 ± 0.0062	0.4291 ± 0.0043
**Cancer**	0.4866 ± 0.0172	0.1732 ± 0.0572	0.3092 ± 0.0441	0.5429 ± 0.0163
	m_{31}	m_{32}	m_{33}	m_{34}
**Normal**	0.4618 ± 0.0037	0.4812 ± 0.0041	0.2818 ± 0.0088	0. 5786 ± 0.0047
**Cancer**	0.3677 ± 0.0257	0.3461 ± 0.0149	0.1743 ± 0.0428	0.4181 ± 0.0839
	m_{41}	m_{42}	m_{43}	m_{44}

**Normal**	0.4521 ± 0.0057	0.4386 ± 0.0087	0.4411 ± 0.0113	0.4667 ± 0.0049
**Cancer**	0.3716 ± 0.0512	0.3509 ± 0.0098	0.3612 ± 0.0135	0.4450 ± 0.0177

Figure 12 shows the t-test results for the intensity differences between the elements of the normal and cancerous colorectal tissues. The element intensities of the healthy samples differ from those of the cancerous samples, with a significance level of $p < 0.0001$ for almost all the elements, including m_{13}, m_{14}, m_{22}, m_{23}, m_{24}, m_{31}, m_{32}, m_{33}, m_{34}, m_{41}, m_{42}, and m_{43}. In other words, the matrix elements of the two tissue classes are statistically different, and hence the polarimetric images provide valid inputs for AI models designed to distinguish between them.

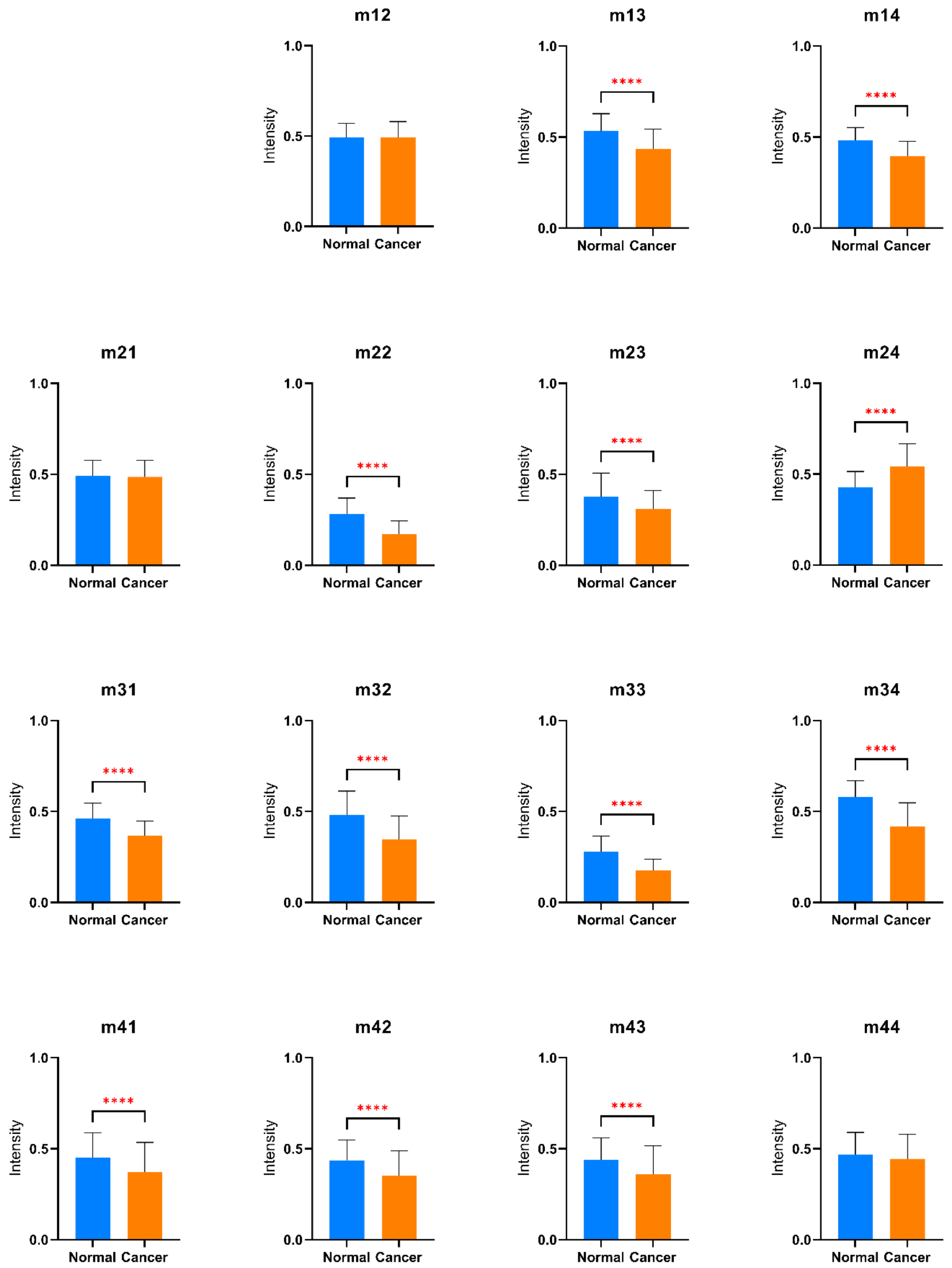

Fig. 12. Average intensity of Mueller matrix elements in normal and cancerous colorectal tissues. T-tests with statistically significant levels of $p < 0.0001$ (marked by ****) are used to examine differences in MMT parameters.

5.1.2 Mueller matrix transformation parameters

One of the main obstacles to the practical application of the Mueller matrix is the lack of obvious physical meaning for each component. In particular, each element may be significantly affected by different

structural traits, which causes the appearance of the Mueller matrix to be very different for different dispersion media. Thus, the concept of MMT parameters has been introduced to provide a more quantitative approach for measuring the polarization variables of the Mueller matrix components associated with specific microstructures or optical characteristics of the medium, such as the subwavelength scatterer density values and widths, or fiber orientation and alignment [25]. In the present study, polarization images were produced using each of the MMT parameters, and the corresponding Mueller matrix was then constructed pixel-by-pixel using Python code to integrate the MMT images. Fig. 13 shows the MMT parameter values obtained from Eqs. (2) – (6) for healthy and malignant colorectal tissues, respectively. Both samples have values of A and G close to 1, which indicates significant anisotropy [25]. The values of b and t for the cancerous tissue are also slightly lower than those for the healthy sample. However, the depolarization power, Δ, and parameter b have an inverse relationship, as discussed by He et al. [25]. Hence, the depolarization power of the malignant sample is higher than that of the benign sample. According to Sun et al. [27], a higher value of Δ indicates a greater anisotropy. Thus, the results presented in Fig. 13 confirm the finding in Fig. 11 that the cancerous tissue sample is more anisotropic than the healthy sample and has a more complex microstructure.

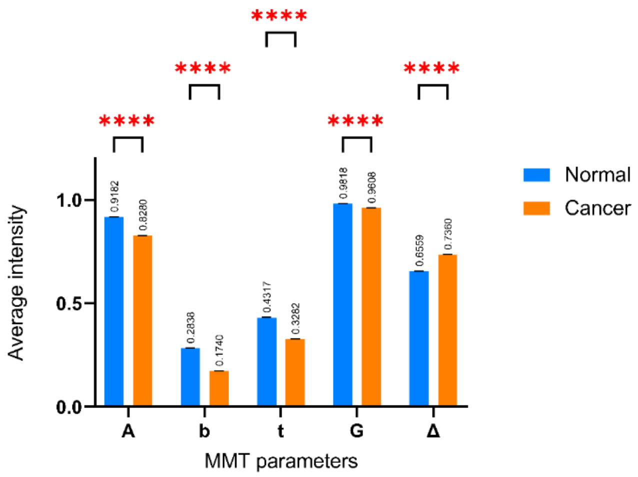

Fig. 13. MMT parameters in normal and cancerous colorectal tissues. The star symbols represent the p-values < .0001 (marked by ****), as determined by paired T-test.

5.1.3 Frequency distribution histograms (FDHs)

Fig. 14 shows the FDHs of the intensity of the 15 normalized elements in the Mueller matrix images of the healthy and cancerous colorectal tissue samples. Although the two curves in each figure overlap, in most cases the peaks of the curves are distinct. Consequently, the intensity feature of the images provides a viable means of differentiating between the two sample classes. However, for each element, the AUCs of the healthy and malignant samples differ, and thus an appropriate setting of the machine learning hyperparameters is essential to determine the elements required to most reliably classify the two groups of data.

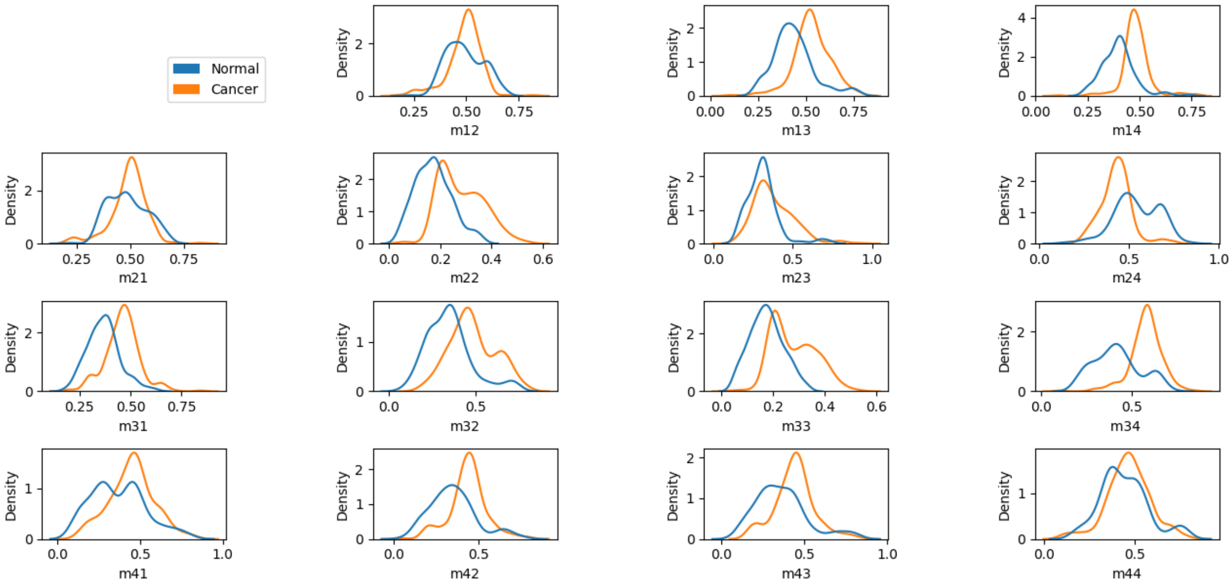

Fig. 14. FDHs of 15 Mueller matrix elements in normal and colorectal cancer tissues.

5.2. AI models for classification of colorectal cancer based on ColoPola dataset

Figure 15 shows the classification performance of the five DL models when applied to the validation and testing sets. The EfficientNetV2 model achieved the highest F1 score of the five models on both datasets (F1 = 0.978 for the validation set and 0.965 for the testing set), and showed a difference of less than 1.5% between the two datasets for all four metrics. The DenseNet model also showed a good performance, with all the metrics having a value higher than 90% for both sets, except for the recall metric for the testing set

(0.895). Meanwhile, the EfficientFormerV2 showed similar performance with the DenseNet on F1 score but the EfficientFormerV2 has higher recall (0.978 and 0.947) and lower precision scores (0.937 and 0.871) on both datasets than the DenseNet. The CNN and CNN_2 models achieved a relatively lower performance, with precision values of 0.862 and 0.847, respectively, for the testing set. Similar to the EfficientFormerV2, the CNN_2 model exhibited a large difference of approximately 6% between the precision scores for the validation set and testing set, respectively.

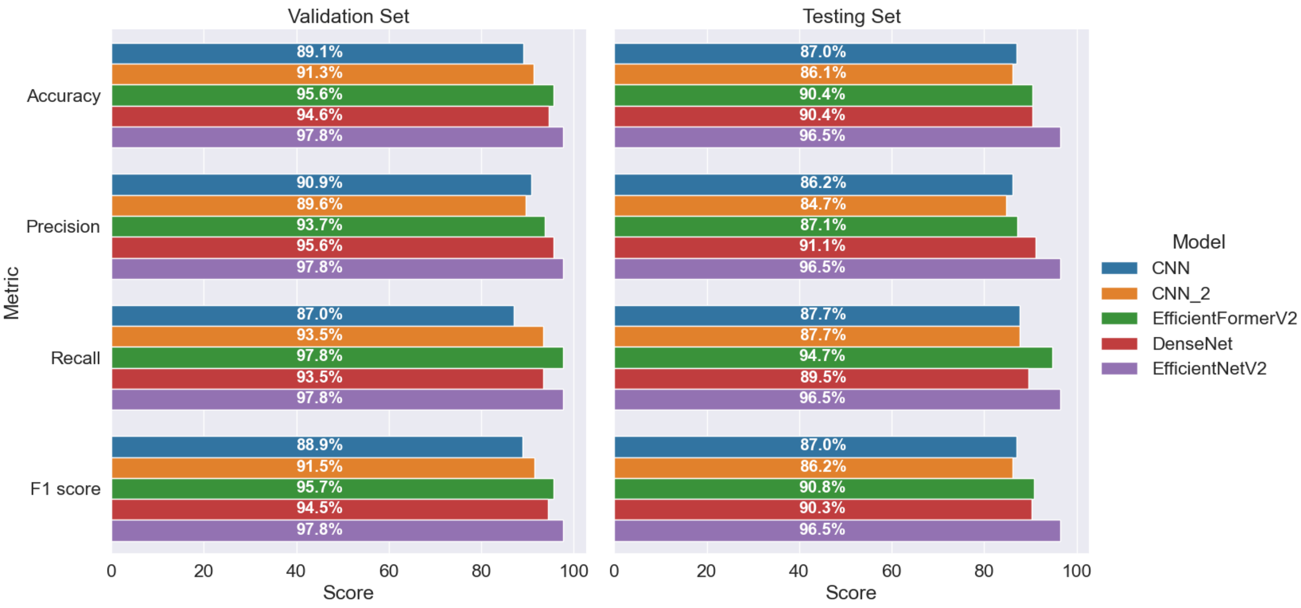

Fig. 15. Performance metrics of five models on validation set and testing set.

As shown in Fig. 16, the CNN model showed a relatively high false positive rate (FPR) when classifying the malignant (positive) cancer class, with three FP samples in the validation set and eight in the testing set. More worryingly, the CNN model also had a high false negative rate (FNR) for the normal (negative) class, with seven FN samples in both datasets. The CNN_2 model exhibited a slightly poorer classification performance, with FP values of five and nine and FN values of three and seven in the validation and testing sets, respectively (Fig. 17). Similar to both CNN and CNN_2, the EfficientFormerV2 model had the high FPR, especially on the testing set with FP values of 8 (Fig. 18). Overall, the CNN model had the lowest recall owing to the high FNR for the two datasets, while the CNN_2 model had the lowest precision score owing to the high FPR of the two datasets. And the EfficientFormerV2 had the

largest difference of precision score between validation and testing sets because of the difference in FP values on these datasets (see Fig. 15).

The DenseNet erroneously classified three cancer samples as normal in the validation dataset, and six cancer samples as normal in the testing dataset (Fig. 19). In contrast, the EfficientNetV2 misclassified only one cancer sample in the validation set and two in the testing dataset (Fig. 20). Overall, therefore, EfficientNetV2 outperformed DenseNet for all four performance metrics, as shown in Fig. 15.

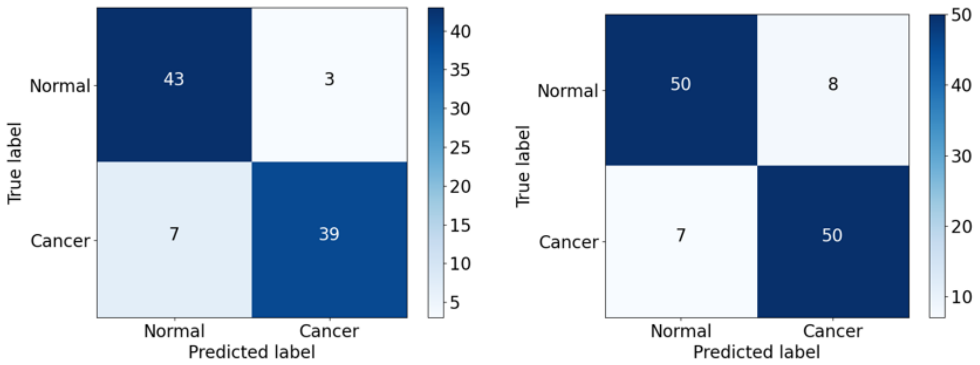

Fig. 16. Confusion matrix for CNN model on validation and testing sets.

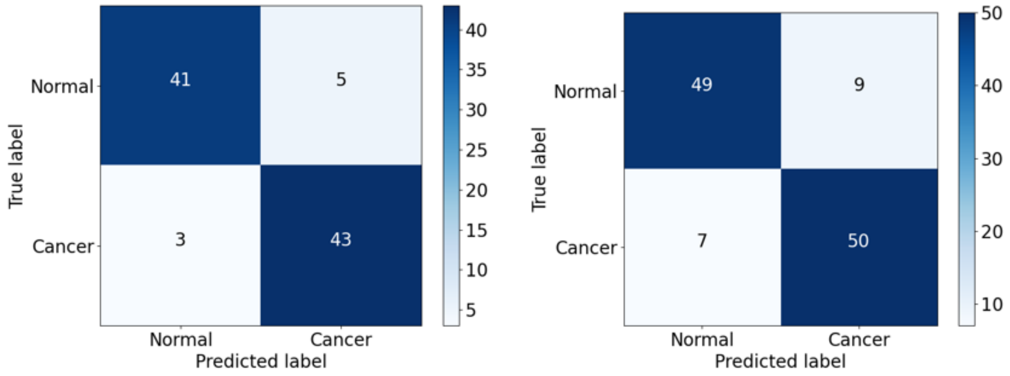

Fig. 17. Confusion matrix for CNN_2 model on validation and testing sets.

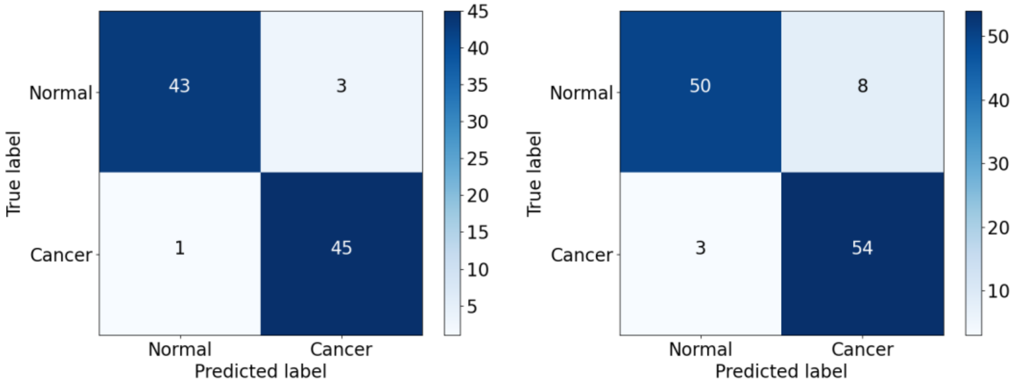

Fig. 18. Confusion matrix for EfficientFormerV2 model on validation and testing sets.

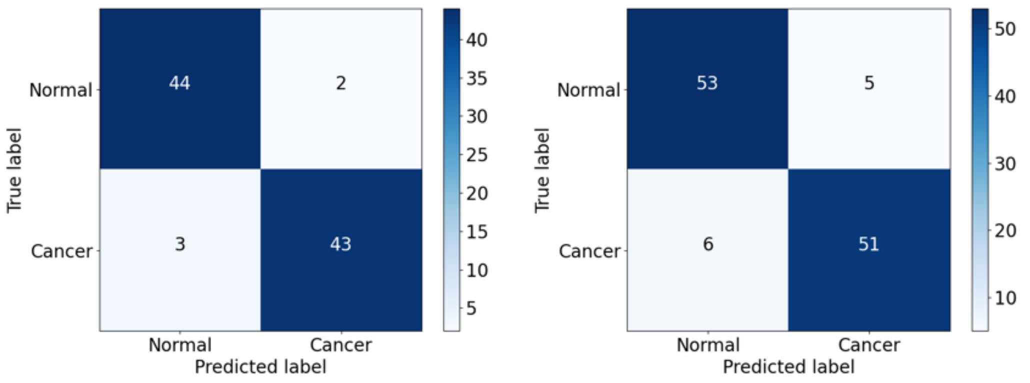

Fig. 19. Confusion matrix for DenseNet model on validation and testing sets.

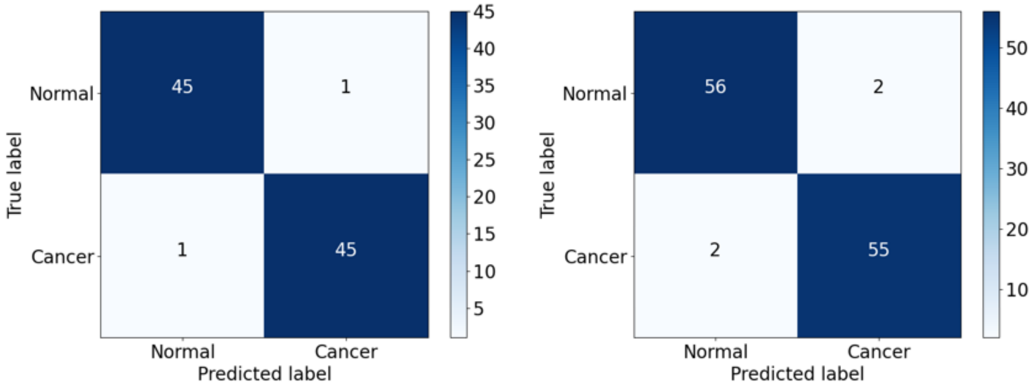

Fig. 20. Confusion matrix for EfficientNetV2 model on validation and testing sets.

Two statistical tests (McNemar's and two-sided binomial) [46] were applied to further compare the binary classification performance of the five trained models when applied to the testing set. The models were compared through side-by-side comparisons under the null hypothesis that the two models should show no significant difference in their classification ability. That is, the number of samples classified as

normal by Model A but classified as cancer by Model B (n_{01}) should be equal to the number of examples classified as cancer by Model A but classified as normal by Model B (n_{10}). The evaluation results are presented in Table 4, where the significance level (α) was set at 0.05 for both statistical tests. In McNemar's test, the *p*-value was calculated based on the chi-square (χ^2) distribution with continuity correction and one degree of freedom. However, some of the paired models (CNN vs. CNN_2, CNN vs. EfficientFormerV2, DenseNet vs. EfficientFormerV2) had very few examples ($n_{01} + n_{10} \leq 10$), and hence a two-sided binomial test was required to properly identify any difference between them. The binomial test for such models was calculated with a probability of 0.5, indicating an assumption that there was a 50% chance of model's output being true or false. The *sig* entries at the foot of the table indicate a significant difference (Yes) if the *p*-value is less than *α*, or no difference (No) if the *p*-value is greater than *α*. Overall, the results presented in the table show no significant difference in the performance of the five trained models on the testing set. In other words, the null hypothesis cannot be rejected, and hence all five models can be used with the ColoPola dataset to detect colorectal cancer.

Table 4. Statistical tests for five trained models on testing set (Do thêm 1 model nữa nên giờ Table 4 này nhiều cột quá. Giờ Table 4 đang được chia thành 2 Tables trong đoạn này, chưa biết gom lại thế nào)

Statistical values	CNN vs. CNN_2	CNN vs. DenseNet	CNN vs. EfficientNetV2	CNN_2 vs. DenseNet	CNN_2 vs. EfficientNetV2	DenseNet vs. EfficientNetV2	CNN vs. EfficientFormerV2	CNN_2 vs. EfficientFormerV2	DenseNet vs. EfficientFormerV2	EfficientNetV2 vs. EfficientFormerV2
n_{01}	5	4	8	5	9	6	5	6	7	9
n_{10}	4	6	9	8	11	5	1	3	1	4
α	0.05	0.05	0.05	0.05	0.05	0.05	0.05	0.05	0.05	0.05
χ^2	0	0.1	0	0.308	0.05	0	1.5	0.444	3.125	1.231
p-value (McNemar)	1	0.752	1	0.579	0.823	1	0.221	0.505	0.077	0.267
p-value (binomial)	1	0.754	1	0.581	0.824	1	0.219	0.508	0.070	0.267
sig	No	No	No	No	No	No	No	No	No	No

Statistical values	CNN vs. CNN_2	CNN vs. DenseNet	CNN vs. EfficientNetV2	CNN_2 vs. DenseNet	CNN_2 vs. EfficientNetV2	DenseNet vs. EfficientNetV2
n_{01}	5	4	8	5	9	6
n_{10}	4	6	9	8	11	5
α	0.05	0.05	0.05	0.05	0.05	0.05
χ^2	0	0.1	0	0.308	0.05	0
p-value (McNemar)	1	0.752	1	0.579	0.823	1
p-value (binomial)	1	0.754	1	0.581	0.824	1
sig	No	No	No	No	No	No

Statistical values	CNN vs. EfficientFormerV2	CNN_2 vs. EfficientFormerV2	DenseNet vs. EfficientFormerV2	EfficientNetV2 vs. EfficientFormerV2
n_{01}	5	6	7	9
n_{10}	1	3	1	4
α	0.05	0.05	0.05	0.05
χ^2	1.5	0.444	3.125	1.231
p-value (McNemar)	0.221	0.505	0.077	0.267
p-value (binomial)	0.219	0.508	0.070	0.267
sig	No	No	No	No

In the present study, the input data had a size of 224×224 and 36 channels, as described in Section 4.1. Based on preliminary experiments, the first convolutional layers in the CNN and CNN_2 models were

designed to extract 128 output features in order to achieve a balance between the amount of usable information obtained and the computational complexity. The original EfficientFormerV2, DenseNet and EfficientNetV2 models are designed to classify color images using the red, green, and blue channel values as the input data. Moreover, the default values of the number of output features in the first convolutional layers of the three models are 16, 64 and 24, respectively. Thus, when processing a 36-channel input, insufficient information may be extracted from the first convolutional layer for transfer to the next layers, particularly in the case of the EfficientFormerV2 and EfficientNetV2 models. However, both DenseNet and EfficientNetV2 models are much deeper and more sophisticated than the CNN and CNN_2 models. Meanwhile, the EfficientFormerV2 has the different strategy when combining the extracted features from convolution and vision transformer networks to obtain the useful information despite of training from scratch. Consequently, they outperform both models despite this potential limitation (see Fig. 15). The performance improvement is particularly evident for the EfficientNetV2 model, owing to its use of various techniques (e.g., new convolutional blocks; a combination of optimized scaling on width, height, and resolution; and a progressive learning technique) to optimize the training speed and parameter efficiency.

Besides, one advantage of these models is that the raw polarimetric images from the ColoPola dataset are processed and fed directly into the AI models without additional processing steps such as calculating the Mueller matrix images [29], extracting optical parameters [47, 48], or combining both data types [49]. However, the input data in the present study has a large size, i.e., 36 channels. In other words, each input requires the pre-processing of 36 polarimetric images, followed by the concatenation of the red channels of these images. This is a time-consuming task, which can require a high-performance computing systems for large datasets. Moreover, when applying the transfer learning technique, it is necessary to modify the first layer of the pretrained models to accommodate the new input format.

6. Conclusion

This study has presented a dataset of colorectal cancer polarimetric images, designated as ColoPola, containing 10,368 instances of healthy colorectal tissue and 10,224 instances of colorectal cancer tissue corresponding to 572 tumor slices (36 polarization images per slice). The observation results have shown that the Mueller matrix images of both classes have diagonal symmetry. However, in cancerous tissues, the diagonal components are generally lower than those in healthy tissues, indicating that the cancerous samples have a more complex microstructure. The difference in the degree of anisotropy between the two sample classes has been confirmed through a comparison of the MMT parameters, which showed that the Δ value of the cancerous samples is higher than that of the healthy samples. Notably, a significant difference has been found between all the MMT parameter values for the two classes. In other words, the MMT parameters provide a viable means of distinguishing between the healthy and malignant CRC samples.

The utility of the ColaPola dataset for classification purposes has been evaluated using five DL models, including three models trained from scratch (CNN, CNN_2 and EfficientFormerV2) and two pretrained models (DenseNet and EfficientNetV2). For each model, the input data had a size of 224×224×36, where the latter dimension corresponds to the red channel values of the 36 polarimetric images associated with each tumor slice. The results showed that EfficientFormerV2, DenseNet and EfficientNetV2 both achieved an F1 score of more than 90% on the testing set. By contrast, the CNN and CNN-2 models achieved lower F1 scores of 87% and 86.2%, respectively. The superior performance of the pretrained models can be attributed to their deeper structures and more sophisticated operations. Overall, the results suggest that the ColoPola dataset serves as a useful resource for further research into the identification of CRC malignant tissue using statistical methods based on the MMT parameters or machine learning methods based on the red channel values of the polarimetric images.

Availability of Supporting Source Code and Requirements

Project name: Colorectal cancer detection

Project homepage: https://github.com/haile493/Colorectal-cancer-detection-using-ColoPola-dataset

Operating system(s): Platform independent

Programming language: Python

License: GNU GPL v3.0

RRID: SCR_024827

Abbreviations

AI: artificial intelligence; AUC: area under the receiver operating characteristic curve; AvgPool: average pooling; BN: batch normalization; CAD: computer-aided detection; CCD: charge-coupled device; CLAHE: contrast limited adaptive histogram equalization; CNN: convolutional neural network; ColoPola: colorectal cancer polarimetric image; Conv: convolutional layer; CRC: colorectal cancer; CT: computed tomography; DL: deep learning; DNA: deoxyribonucleic acid; DNN: deep neural network; FC: fully connected; FDH: frequency distribution histogram; FN: false negative; FP: false positive; H&E: Hematoxylin and Eosin; MaxPool: max pooling; MMT: Mueller matrix transformation; ML: machine learning; MRI: magnetic resonance imaging; PSA: polarization state analyzer; RAR: Roshal archive; ReLU: rectified linear unit; RGB: red, green and blue; RNN: recurrent neural network; SSL: semi-supervised learning; TN: true negative; TP: true positive.

Author Contributions

Conceptualization, T.T.H.P.; methodology, T.T.H.P. and T.H.L.; validation, T.V.N., T.H.N., Q.H.P., T.H.L., and T.T.H.P.; formal analysis and investigation, T.V.N., T.H.N., Q.H.Q.V., Q.H.P., T.H.L., and T.T.H.P.; resources, T.V.N., T.H.N., T.H.L., and T.T.H.P.; data curation, T.V.N., T.H.N., Q.H.Q.V., T.H.L., and T.T.H.P.; writing – original draft preparation, T.T.H.P., Q.H.Q.V., T.V.N., T.H.N., Q.H.P., and T.H.L.; writing – review and editing, T.T.H.P., Q.H.P., and T.H.L.; visualization, T.T.H.P., and T.H.L.;

software, T.H.L.; supervision, T.T.H.P. and T.H.L.; project administration, T.T.H.P.; funding acquisition, T.T.H.P. All authors have read and agreed to the published version of the manuscript.

Declaration of Competing Interest

The authors declare that they have no relevant financial interests in the manuscript and no other potential conflicts of interest.

Funding

This study was supported by Vietnam National University Ho Chi Minh City (VNU-HCM) under Grant No. DS2023-28-02. The funders had no role in study design, data collection and analysis, decision to publish, or preparation of the manuscript.

Data availability

The dataset supporting the results of this article is available in the Zenodo repository [22, 29].

References

1. Sung H, et al. Global Cancer Statistics 2020: GLOBOCAN Estimates of Incidence and Mortality Worldwide for 36 Cancers in 185 Countries. CA Cancer J Clin 2021;71(3):209-249. https://doi.org/10.3322/caac.21660
2. Sninsky JA, et al. Risk factors for colorectal polyps and cancer. Gastrointest Endosc Clin N Am 2022;32(2):195-213. https://doi.org/10.1016/j.giec.2021.12.008
3. Bond JH. Colorectal cancer screening: the potential role of virtual colonoscopy. J Gastroenterol 2002;37 Suppl 13:92-6. https://doi.org/10.1007/BF02990108
4. Akhtar R, Lee M, and Itzkowitz SH. Colonoscopy versus computed tomography colonography for colorectal cancer screening. Mt Sinai J Med 2010;77(2):214-24. https://doi.org/10.1002/msj.20175
5. Bretthauer M, Holme O, and Garborg K. Computed tomography colonography vs. colonoscopy for colorectal cancer screening: close call, but not closed case. Endoscopy 2013;45(3):159-60. https://doi.org/10.1055/s-0032-1326208
6. Le NT, and Dao HV. Colorectal cancer in Viet Nam. Colorectal Cancer. IntechOpen, Jul. 14, 2021. https://doi.org/10.5772/intechopen.93730
7. Winawer SJ, et al. Prevention of Colorectal Cancer by Colonoscopic Polypectomy. N Engl J Med 1993;329:1977-1981. https://doi.org/10.1056/NEJM199312303292701
8. Ahlquist DA. Stool-based tests vs screening colonoscopy for the detection of colorectal cancer. Gastroenterol Hepatol (N Y) 2019;15(8):437-440. https://pubmed.ncbi.nlm.nih.gov/31592245/
9. Kamiya K, et al. Long-term effects of radiation exposure on health. Lancet 2015;386(9992):469-78. https://doi.org/10.1016/S0140-6736(15)61167-9
10. Taqi AH, Faraj KA, and Zaynal SA. The effect of long-term X-ray exposure on human lymphocyte. J Biomed Phys Eng 2019;9(1):127-132. https://pubmed.ncbi.nlm.nih.gov/30881942/
11. Schmitt JM, Gandjbakhche AH, and Bonner RF. Use of polarized light to discriminate short-path photons in a multiply scattering medium. Appl Opt 1992;31(30):6535-46. https://doi.org/10.1364/AO.31.006535
12. Jacques SL, et al. Polarized light transmission through skin using video reflectometry: toward optical tomography of superficial tissue layers. In Lasers in Surgery: Advanced Characterization, Therapeutics, and Systems VI 1996;2671:199-210. https://doi.org/10.1117/12.240009
13. Demos SG, and Alfano RR. Optical polarization imaging. Appl Opt 1997;36(1):150-5. https://doi.org/10.1364/AO.36.000150

14. Mourant JR, et al. Scattering properties of biological cells. In Biomedical Optical Spectroscopy and Diagnostics / Therapeutic Laser Applications, E. Sevick-Muraca and J. Izatt, eds., Vol. 22 of OSA Trends in Optics and Photonics (Optica Publishing Group, 1998), paper BMA4. https://doi.org/10.1364/BOSD.1998.BMA4

15. Kim SH, et al. Computer-aided detection of colonic polyps at CT colonography using a Hessian matrix-based algorithm: preliminary study. AJR Am J Roentgenol 2007;189(1):41-51. https://doi.org/10.2214/AJR.07.2072

16. Chen PJ, et al. Accurate classification of diminutive colorectal polyps using computer-aided analysis. Gastroenterology 2018;154(3):568-575. https://doi.org/10.1053/j.gastro.2017.10.010

17. Thakur N, Yoon H, and Chong Y. Current trends of artificial intelligence for colorectal cancer pathology image analysis: A systematic review. Cancers (Basel) 2020;12(7):1884. https://doi.org/10.3390/cancers12071884

18. Xu L, et al. Colorectal cancer detection based on deep learning, J Pathol Inform 2020;11(1):28. https://doi.org/10.4103/jpi.jpi_68_19

19. Iizuka O, et al. Deep learning models for histopathological classification of gastric and colonic epithelial tumours. Sci Rep 2020;10(1):1504. https://doi.org/10.1038/s41598-020-58467-9

20. Yu G, et al. Accurate recognition of colorectal cancer with semi-supervised deep learning on pathological images, Nat Commun 2021;12:6311. https://doi.org/10.1038/s41467-021-26643-8

21. Tharwat M, et al. Colon cancer diagnosis based on machine learning and deep learning: Modalities and analysis techniques, Sensors 2022;22(23):9250. https://doi.org/10.3390/s22239250

22. Pham TTH, et al. A dataset of colorectal cancer histopathological images (V1.0) [Data set]. Zenodo. 2024. https://doi.org/10.5281/zenodo.14237234

23. Liu B, et al. Mueller polarimetric imaging for characterizing the collagen microstructures of breast cancer tissues in different genotype. Opt Commun 2019;433:60-67. https://doi.org/10.1016/j.optcom.2018.09.037

24. He H, et al. A possible quantitative Mueller matrix transformation technique for anisotropic scattering media/Eine mögliche quantitative Müller-Matrix-Transformations-Technik für anisotrope streuende Medien. Photonics Lasers Med 2013;2(2):129-137. https://doi.org/10.1515/plm-2012-0052

25. He C, et al. Characterizing microstructures of cancerous tissues using multispectral transformed Mueller matrix polarization parameters. Biomed Opt Express 2015;6(8):2934-45. https://doi.org/10.1364/BOE.6.002934

26. Nan Z, et al. Linear polarization difference imaging and its potential applications. Appl Opt 2009;48(35):6734-9. https://doi.org/10.1364/AO.48.006734

27. Sun M, et al. Characterizing the microstructures of biological tissues using Mueller matrix and transformed polarization parameters. Biomed Opt Express 2014;5(12):4223-34. https://doi.org/10.1364/BOE.5.004223

28. Guo Y, et al. A study on forward scattering Mueller matrix decomposition in anisotropic medium. Opt Express 2013;21(15):18361-70. https://doi.org/10.1364/OE.21.018361

29. Le HM, et al. Mueller matrix imaging polarimetry technique for dengue fever detection. Opt Commun 2022;502:127420. https://doi.org/10.1016/j.optcom.2021.127420

30. Pham TTH, et al. ColoPola: A dataset of colorectal cancer polarimetric images (Mueller matrix elements) for colorectal cancer detection (V1.2) [Data set]. Zenodo. 2023. https://doi.org/10.5281/zenodo.10068018

31. Pham TTH, et al. Combined Mueller matrix imaging and artificial intelligence classification framework for Hepatitis B detection. J Biomed Opt 2022;27(7):075002. https://doi.org/10.1117/1.JBO.27.7.075002

32. Ioffe S, and Szegedy C. Batch normalization: accelerating deep network training by reducing internal covariate shift. In Proceedings of the 32nd International Conference on International Conference on Machine Learning (ICML2015) 2015;37:448–456. Accessed 02 June 2023, https://arxiv.org/abs/1502.03167

33. Srivastava N, et al. Dropout: a simple way to prevent neural networks from overfitting. J Mach Learn Res 2014;15(1):1929–1958. Accessed 02 June 2023, http://jmlr.org/papers/v15/srivastava14a.html

34. Simonyan K, and Zisserman AJC. Very deep convolutional networks for large-scale image recognition. The 3rd International Conference on Learning Representations (ICLR2015). Accessed 02 June 2023, https://arxiv.org/abs/1409.1556

35. Springenberg J, et al. Striving for simplicity: the all convolutional net. The 3rd International Conference on Learning Representations (ICLR2015). Accessed 02 June 2023, https://arxiv.org/abs/1412.6806

36. Li Y, et al. Rethinking vision transformers for mobilenet size and speed. In 2023 IEEE/CVF International Conference on Computer Vision (ICCV) 2023;16843-16854. https://doi.org/10.1109/ICCV51070.2023.01549

37. Li Y, et al. EfficientFormer: Vision transformers at MobileNet speed. NIPS'22: Proceedings of the 36th International Conference on Neural Information Processing Systems 2022;12934 - 12949.

38. Huang G, et al. Densely connected convolutional networks. 2017 IEEE Conference on Computer Vision and Pattern Recognition (CVPR) 2017;2261-2269. https://doi.org/10.1109/CVPR.2017.243.

39. Tan M, and Le QV. EfficientNetV2: Smaller Models and Faster Training. In Proceedings of the 38th International Conference on Machine Learning, M. Marina and Z. Tong, Editors. 2021, PMLR: Proceedings of Machine Learning Research 2021;10096--10106. Accessed 21 June 2023, https://arxiv.org/abs/2104.00298

40. Tan M, and Le QV. EfficientNet: Rethinking Model Scaling for Convolutional Neural Networks. In Proceedings of the 36th International Conference on Machine Learning, C. Kamalika and S. Ruslan, Editors. 2019, PMLR:

Proceedings of Machine Learning Research 2019;6105--6114. Accessed 21 June 2023, https://arxiv.org/abs/1905.11946

41. He K, et al. Delving deep into rectifiers: surpassing human-level performance on Imagenet classification. 2015 IEEE International Conference on Computer Vision (ICCV) 2015;1026-1034. https://doi.org/10.1109/ICCV.2015.123

42. Prechelt L. Early stopping-But when? In: Neural Networks: Tricks of the trade, Springer, 1998, 55–69. https://doi.org/10.1007/978-3-642-35289-8_5

43. Loshchilov I, and Hutter F. Decoupled weight decay regularization. 7th International Conference on Learning Representations (ICLR2019) 2019, LA, USA. Accessed 15 March 2023, https://arxiv.org/abs/1711.05101

44. Buslaev A, et al. Albumentations: fast and flexible image augmentations. Information 2020;11(2):125. https://doi.org/10.3390/info11020125

45. Thi-Thu-Hien Pham, Thao-Ngan Ngoc Quach, and Quoc-Hoang-Quyen Vo "Analysis of polarization features of human breast cancer tissue by Mueller matrix visualization," Journal of Biomedical Optics 2024; 29(5), 052917. https://doi.org/10.1117/1.JBO.29.5.052917

46. Dietterich TG. Approximate statistical tests for comparing supervised classification learning algorithms. Neural Comput 1998;10(7):1895-1923. https://doi.org/10.1162/089976698300017197

47. Luu TN, et al. Characterization of Mueller matrix elements for classifying human skin cancer utilizing random forest algorithm, J Biomed Opt 2021;26(7):075001. https://doi.org/10.1117/1.JBO.26.7.075001

48. Luu TN, et al. Classification of human skin cancer using Stokes-Mueller decomposition method and artificial intelligence models. Optik 2022;249:168239. https://doi.org/10.1016/j.ijleo.2021.168239

49. Pham TTH, et al. Polarimetric imaging combining optical parameters for classification of mice non-melanoma skin cancer tissue using machine learning. Heliyon 2023;9(11):e22081. https://doi.org/10.1016/j.heliyon.2023.e22081
