# Supplementary material for: ColoPola: A polarimetric imaging dataset for colorectal cancer detection
Source: Gigascience. 2025 Oct 16;14:giaf120. doi: 10.1093/gigascience/giaf120 (PMC12530094; doi:10.1093/gigascience/giaf120)
Supplement: giaf120_Reviewer_1_Report_Original_Submission — Ruitian Gao -- 6/29/2025 [file giaf120_reviewer_1_report_original_submission.pdf]

Reviewer Report

Title: ColoPola: A polarimetric imaging dataset for colorectal cancer detection

Version: Original Submission **Date:** 6/29/2025

Reviewer name: Ruitian Gao

Reviewer Comments to Author:

The authors proposed a novel colorectal cancer polarized image dataset named ColoPola, aiming to advance research in this field. The dataset comprises 572 sample slices (288 healthy and 284 malignant), with each slice providing 36 polarized images corresponding to different polarization states, totaling 20,592 images. The authors not only conducted statistical analysis on Mueller matrix elements and Mueller matrix transformation (MMT) parameters of healthy and cancerous tissue samples using Mueller matrix imaging but also identified significant differences between the two groups, confirming the feasibility of MMT parameters in distinguishing healthy and malignant CRC samples. Additionally, they evaluated the dataset's utility for classification tasks using five deep learning models (CNN, CNN_2, EfficientFormerV2, DenseNet, and EfficientNetV2). The results demonstrated that the EfficientNetV2 model achieved the best performance, with an F1-score of 0.965 on the test set, and all evaluation metrics exceeded 0.95. The study suggests that the ColoPola dataset holds great potential as a polarization-based optical imaging tool for CRC diagnosis in clinical practice. However, I believe there are still some issues that need to be clarified before recommending publication. The authors should address the following points in their revision:
Major comments:
1) The study uses only the red channel as input, noting that it contains full intensity values in most images, while green and blue channels have near-zero or low-range values. However, this risks losing potential information from other channels. Although the authors justify this choice for consistent learning performance between healthy and malignant samples, they do not fully analyze whether multi-channel data could improve feature extraction. A deeper discussion on the trade-offs of single-channel vs. multi-channel approaches would strengthen the methodology.
2) The study presents the first polarized image dataset for colorectal cancer, positioning it as filling an important gap in biomedical imaging. However, the claimed diagnostic superiority over conventional H&E-stained pathology requires stronger evidence. While the technical methodology is comprehensively described, the manuscript would benefit from addressing key clinical implementation factors including cost analysis, processing speed, and integration with standard pathology workflows. A more detailed comparison with existing diagnostic methods would better demonstrate the potential clinical impact of this technology.
3) The study's exclusive use of samples from two Vietnamese hospitals may introduce geographic or ethnic biases that require further discussion. Additionally, the dataset composition lacks clear documentation regarding cancer stage distribution and inclusion of confounding samples (e.g., benign polyps), potentially compromising the evaluation of model generalizability. These limitations should be explicitly addressed to properly contextualize the findings.

4) The provided code lacks proper documentation, with unclear functionality for individual scripts, compromising reproducibility. We recommend enhancing the README.md file with detailed descriptions of each script's purpose and execution workflow to facilitate replication of results.
5) The description of Mueller matrix transformation in the Methods section requires clearer and more logical presentation. We recommend restructuring this section with step-by-step mathematical formulations and explicit interpretations to improve conceptual clarity.
Minor comments:
1) Several figures contain small text, compromising readability. Some fonts appear unprofessional and contain irregular spacing within words (e.g. Fig. 5). We recommend verifying all figure fonts by printing the manuscript on A4 paper and optimizing the visual presentation accordingly.
2) To ensure robust evaluation, we recommend assessing model stability through multiple randomized data splits beyond the single partition reported in Table 1.
3) In Fig. 2, "Collecting 36 image data â†’ using Python to process to 16 images", the manuscript should clarify the rationale and methodology for reducing the processed images from 36 to 16 in Python, as this critical preprocessing step currently lacks justification and may confuse readers.
4) In Line 278 "Two pretrained DL models, DenseNet-121 and EfficientNetV2-M, were selected and finetuned for the ColoPola dataset." The pretraining details should be specified.
5) In Line 294 "As shown, most of the hyperparameters were the same for all five models." How were the hyperparameters determined, particularly given that "Models from scratch" and "Pretrained models" share identical settings in Table 2? For scratch-trained models, were additional epochs considered to ensure sufficient convergence?
6) The manuscript requires thorough proofreading to address:
(i) Typographical errors (e.g., "sox" in Line 155, unformatted "m11" in Line 334 vs. properly subscripted instances);
(ii) Text corruption (Lines 448-449);
(iii) Inconsistent figure references ("Figure X" vs. "Fig. X").

Methods

Are the methods appropriate to the aims of the study, are they well described, and are necessary controls included? Choose an item.

Conclusions

Are the conclusions adequately supported by the data shown? Choose an item.

Reporting Standards

Does the manuscript adhere to the journal's guidelines on minimum standards of reporting? Choose an item.

Choose an item.

Statistics

Are you able to assess all statistics in the manuscript, including the appropriateness of statistical tests used? Choose an item.

Quality of Written English

Please indicate the quality of language in the manuscript: Choose an item.

Declaration of Competing Interests

Please complete a declaration of competing interests, considering the following questions:

- Have you in the past five years received reimbursements, fees, funding, or salary from an organisation that may in any way gain or lose financially from the publication of this manuscript, either now or in the future?
- Do you hold any stocks or shares in an organisation that may in any way gain or lose financially from the publication of this manuscript, either now or in the future?
- Do you hold or are you currently applying for any patents relating to the content of the manuscript?
- Have you received reimbursements, fees, funding, or salary from an organization that holds or has applied for patents relating to the content of the manuscript?
- Do you have any other financial competing interests?
- Do you have any non-financial competing interests in relation to this paper?

If you can answer no to all of the above, write 'I declare that I have no competing interests' below. If your reply is yes to any, please give details below.

I declare that I have no competing interests

I agree to the open peer review policy of the journal. I understand that my name will be included on my report to the authors and, if the manuscript is accepted for publication, my named report including any attachments I upload will be posted on the website along with the authors' responses. I agree for my report to be made available under an Open Access Creative Commons CC-BY license (http://creativecommons.org/licenses/by/4.0/). I understand that any comments which I do not wish to be included in my named report can be included as confidential comments to the editors, which will not be published.

Choose an item.

To further support our reviewers, we have joined with Publons, where you can gain additional credit to further highlight your hard work (see: https://publons.com/journal/530/gigascience). On publication of this paper, your review will be automatically added to Publons, you can then choose whether or not to claim your Publons credit. I understand this statement.

Yes Choose an item.
